# *Bacteroides dorei* BDX-01 alleviates DSS-induced experimental colitis in mice by regulating intestinal bile salt hydrolase activity and the FXR-NLRP3 signaling pathway

**DOI:** 10.3389/fphar.2023.1205323

**Published:** 2023-05-24

**Authors:** Xiaowei Sun, Zhenhui Chen, Lu Yu, Weisen Zeng, Boyuan Sun, Hongying Fan, Yang Bai

**Affiliations:** ^1^ Guangdong Provincial Key Laboratory of Gastroenterology, Department of Gastroenterology, Institute of Gastroenterology of Guangdong Province, Nanfang Hospital, Southern Medical University, Guangzhou, China; ^2^ Guangdong Provincial Key Laboratory of Tropical Disease Research, Department of Microbiology, School of Public Health, Southern Medical University, Guangzhou, China; ^3^ Department of Radiation Oncology, Nanfang Hospital, Southern Medical University, Guangzhou, China; ^4^ Department of Cell Biology, School of Basic Medical Sciences, Southern Medical University, Guangzhou, China; ^5^ The Second School of Clinical Medicine, Southern Medical University, Guangzhou, China

**Keywords:** *Bacteroides*, bile acid, bile salt hydrolase, farnesoid X receptor, inflammasome, probiotic, ulcerative colitis

## Abstract

**Background:** The relationships among intestinal dysbiosis, bile acid (BA) metabolism disorders, and ulcerative colitis pathogenesis are now recognized. However, how specific strains regulate BA metabolism to alleviate colitis is still unclear. This study investigated the effects of *Bacteroides dorei* on the development of acute colitis and elucidated the underlying mechanisms.

**Methods:** The safety of BDX-01 was evaluated *in vitro* and *in vivo*. 2.5% dextran sulfate sodium (DSS) induced colitis in C57BL/6 mice, Caco-2, and J774A.1 cells were used to evaluate the anti-inflammatory effect of BDX-01. qPCR and Western blotting were used to detect the expression of inflammatory pathways. Microbiota composition was analyzed by 16S rRNA gene sequencing. Enzyme activity analysis and targeted metabolomics were used to analyze fecal bile salt hydrolase (BSH) and BA levels. Antibiotic-induced pseudo-germ-free mice were used to investigate the role of gut microbiota in the alleviation of colitis by BDX-01.

**Results:** We confirmed the safety of novel strain *Bacteroides dorei* BDX-01 *in vitro* and *in vivo*. Oral BDX-01 administration significantly ameliorated the symptoms and pathological damage of DSS-induced acute colitis. Moreoever, 16S rRNA sequencing and enzyme activity analysis showed that BDX-01 treatment increased intestinal BSH activity and the abundance of bacteria harboring this enzyme. Targeted metabolomics revealed that BDX-01 significantly increased intestinal BA excretion and deconjugation. Certain BAs act as FXR agonists. The β-muricholic acid (βMCA): taurine β-muricholic acid (T-βMCA) and cholic acid (CA): taurocholic acid (TCA) ratios and the deoxycholic acid (DCA) level decreased markedly in the colitis models but increased substantially in BDX-01-treated mice. The colonic farnesoid X receptor (FXR) and fibroblast growth factor 15 (FGF15) were upregulated in mice treated with BDX-01. BDX-01 downregulated the expression of colonic proinflammatory cytokines pyrin domain-containing 3 (NLRP3), ASC, cleaved caspase-1, and IL-1β. Antibiotic treatment didn’t abolish the protective effect of BDX-01 on colitis. *In vitro* studies showed TβMCA abolished the effects of BDX-01 on FXR activation and inhibition of the NLRP3 inflammasome activation.

**Conclusion:** BDX-01 improved DSS-induced acute colitis by regulating intestinal BSH activity and the FXR-NLRP3 signaling pathway. Our findings indicate that BDX-01 is a promising probiotic to improve the management of ulcerative colitis.

## 1 Introduction

The onset and progression of inflammatory bowel disease (IBD) are affected by several complex factors (Abraham and Cho, 2009). The contributions of heredity and environment to IBD are well studied. However, relatively little is known about the impact of the gut microbiota on this disorder (Ni et al., 2017; Pittayanon et al., 2020). IBD includes two types: ulcerative colitis (UC) and Crohn’s disease (CD), both of which are associated with gut dysbiosis that occurs after the intestinal microbiota undergoes compositional and metabolic changes (Ni et al., 2017; Shen et al., 2018). Gut microbiota disturbances increase the content of harmful bacteria and dysregulate gut homeostasis (Shen et al., 2018). Bile acids (BAs) are microbiota-associated metabolites that regulate intestinal homeostasis and inflammation in UC (Kriaa et al., 2022). BA metabolism is defective in patients with IBD, as indicated by increased levels of primary BAs (PBAs) and/or their conjugated forms, accompanied by decreased levels of second BAs (SBAs) (Lavelle and Sokol, 2020; Biagioli et al., 2021; Gallagher et al., 2021). The gut microbiota of patients with IBD is characterized by low BSH activity (Ogilvie and Jones, 2012; Duboc et al., 2013).

BA is synthesized in the liver and then secreted into the intestine, where most of it is reabsorbed in the terminal ileum and finally transported back to the liver by portal blood. Through this process, BA functions as a signaling molecule by acting on nuclear and membrane receptors in the liver and intestine. Farnesoid X receptor (FXR) is a member of the steroid/thyroid hormone receptor family of ligand-activated transcription factors (Gomez-Ospina et al., 2016). As one of the nuclear receptors of BA, FXR inhibits BA synthesis by decreasing the expression of cytochrome p450 enzyme cholesterol 7α-hydroxylase (CYP7A1) through a small heterodimer partner (SHP) in the liver. In the ileum, FXR participates in BA reabsorption by regulating the expression of BA transporters and induces the secretion of fibroblast growth factor-19/15, which is delivered to the liver via the portal vein to suppress CYP7A1 expression (Inagaki et al., 2005).

The gut microbiota is critical for BA metabolism and converts conjugated primary BAs into secondary BAs via deconjugation of BSH and epimerization of 7α/β-dehydroxylase. BA transformation by BSH-competent bacteria determines the magnitude of BA-induced nuclear FXR activation in the gut (Long et al., 2017; Sun et al., 2018). Chenodeoxycholic acid (CDCA) is the major activator of FXR, followed by deoxycholic acid (DCA), colic acid (LCA), and cholic acid (CA) (Jones et al., 2014). The gut microbiota may inhibit the synthesis of BAs by decreasing the level of TβMCA, an FXR antagonist (Sayin et al., 2013). FXR is implicated in intestinal immunomodulation and barrier function (Vavassori et al., 2009). Activation of FXR inhibits intestinal inflammation and protects the intestinal barrier in IBD (Gadaleta et al., 2011).

*Bacteroides dorei* is prevalent in the human gut microbiota and can inhibit the growth of the colonic pathogen *Clostridium difficile* (Anonye et al., 2019), improve host resistance to influenza virus infection (Song et al., 2021), mitigate atherosclerosis, and suppress proinflammatory cytokine production (Yoshida et al., 2018). *B. Dorei* possesses high BSH activity *in vitro* (Yao et al., 2018), and its metabolites regulate the expression of genes involved in FXR and BA homeostasis in a gene- and tissue-specific manner in diet-induced obese mice (Zhang et al., 2015). These studies suggest that it may have regulatory effects on bile acid metabolism and anti-inflammatory effects.

Here, we isolated a novel probiotic candidate strain *B. dorei* BDX-01 from the feces of a healthy human subject. This study aimed to confirm the effects of *B. dorei* BDX-01 on the symptoms and progression of UC and elucidate the molecular mechanisms involved. *B. dorei* BDX-01 treatment was also compared with mesalazine, a first-line agent in patients with active UC (Magro et al., 2020).

## 2 Materials and methods

### 2.1 *B. dorei* strain BDX-01 isolation and molecular identification

The candidate probiotic *B. dorei* BDX-01 was isolated in a stool specimen from a healthy human. The sample was inoculated onto brain heart immersion (BHI, Solarbio Life Sciences, Beijing, China) agar medium supplemented with 5% (v/v) defibrotic sheep blood (Solarbio Life Sciences, Beijing, China) and cultured in a 5% CO_2_ anaerobic incubator (No. DG250; Don Whitley Scientific, Bingley, West Yorkshire, United Kingdom) at 37°C for 48 h. The isolated strain had morphophysiological characteristics resembling those of *B. dorei*, according to Bergey’s Manual of Systematic Bacteriology (Krieg et al., 2001). Genomic DNA was extracted from BDX-01 using a DNA extraction kit (Qiagen, Hilden, Germany). 16S rRNA genes were amplified by PCR using 27F (5′-AGA​GTT​TGA​TCC​TGG​CTC​AG-3′) and 1492R (5′-GGT​TAC​CTT​GTT​ACG​ACT​T-3′) as universal primers. Amplified products were sequenced, and 16S rRNA gene sequences were compared with those of various *Bacteroides* strains using BLAST software and the NCBI database (https://www.ncbi.nlm.nih.gov).

### 2.2 Genome sequencing, assembly, and analysis

Total DNA was extracted from strain BDX-01 and the complete genome sequence was determined using an Illumina Hiseq 2,000 sequencing system by Biomarker Technologies. For genome assembly, The filtered reads were assembled by Canu v1.5 software, and then circlator v1.5.5 was taken to cyclizing assembly genome. For genome component prediction, Coding genes prediction was performed by Prodigal v2.6.3. The GenBlastA v1.0.4 program was used to scan the whole genomes after masking predicted functional genes. Putative candidates were then analyzed by searching for non-mature mutations and frame-shift mutations using GeneWise v2.2.0.

Putative virulence factors and antibiotic resistance genes were annotated by BLAST analysis with genes in the Virulence Factors of Pathogenic Bacteria Database (VFDB) (Chen et al., 2005) and Comprehensive Antibiotic Resistance Database (CARD) (Jia et al., 2017), based on a minimum of 50% and 35% amino acid homology, respectively. A BLAST comparison was performed between the genome sequences of strain BDX-01 and *B. dorei* strain 175 (GenBank accession number NZ_CP046176.1). The average nucleotide identity (ANI) was calculated using CJ Bioscience’s online Average Nucleotide Identity (ANI) calculator (https://www.ezbiocloud.net/tools/ani).

### 2.3 Antibiotic resistance testing

The antibiotic resistance breakpoints of *B. dorei* BDX-01 and the type strain *B. dorei* 175 were determined using 14 antibiotics with various antimicrobial mechanisms. The antibiotics included ampicillin, vancomycin, cefoperazone, and meropenem (cell wall synthesis inhibitors); clindamycin, clarithromycin, chloromycetin, tetracycline, streptomycin, and gentamicin (protein synthesis inhibitors); metronidazole, ciprofloxacin, and trimethoprim (nucleic acid synthesis inhibitors); and polymyxin B (cytoplasmic function inhibitor). Overnight 100 μL BDX-01 cultures (10^7^ CFU/mL) were co-cultured anaerobically in sterile 96-well plates (NEST, Wuxi, China) with antibiotics serially diluted between 0.125 and 512 μg/mL. OD_600_ were determined with a microplate reader (Infinite200PRO; Tecan Austria GmbH, Grödig, Austria). The minimal inhibitory concentration (MIC) of different antibiotics was recorded. This metric indicates the lowest doses that inhibits 90% growth of the tested *B. dorei* strains (D’Aimmo et al., 2007). All experiments were performed in biological triplicate.

### 2.4 Cell culture study

The Caco-2 human colon, IEC-6 rat intestinal epithelial and macrophage J774A.1 cell lines were purchased from the American Type Culture Collection (ATCC, Manassas, VA, United States). All cell lines were cultured in Dulbecco’s modified Eagle’s medium (DMEM, Gibco, United States) supplemented with 10% (v/v) fetal bovine serum (FBS, Nobimpex, South America) at 37°C and under a 5% CO_2_ atmosphere.

*In vitro* BDX-01 safety was determined in Caco-2 and IEC-6 cells using a lactate dehydrogenase (LDH) activity assay (Solarbio Life Sciences, Beijing, China). The Caco-2 and IEC-6 cells were inoculated in 96-well plates at a density of 5 × 10^4^/well and incubated at 37°C overnight. Each well contained 100 μL bacterial suspension at 5 × 10^7^ CFU/mL (MOI = 100) or 1 × 10^8^ CFU/mL (MOI = 200). After co-incubation at 37°C under a 5% (v/v) CO_2_ atmosphere for 4 h, the LDH content was determined by microplate analyzer at 572 nm and the cytotoxicity was calculated as follows:

$$cytotoxicity\left(\%\right)=\left[\left(sample–background\right)/total\;LDH\;release\right]\times 100$$

Total LDH was released by lysing the cells in 1% (v/v) TritonX-100.

Before *in vitro* anti-inflammatory assays, Caco-2 cells were seeded in six-well culture plates (NEST, Wuxi, China) and the medium was changed every other day for 14 days to allow complete differentiation. BDX-01 culture (1 × 10^7^ CFU/mL), GW4064 (10 μM), and TβMCA (100 μM) were added to the culture plates, and the suspensions were incubated at 37°C for 12 h. Culture suspensions were removed from each well and cells were then treated with 100 ng/mL TNF-α for 4 h. J774A.1 cells were seeded in six-well culture plates and incubated at 37°C overnight. GW4064 (10 μM) and TβMCA (100 μM) were added to the culture plates, and the suspensions were incubated at 37°C for 12 h. The culture suspensions were removed from each well and a new medium was added according to different groups. For BDX-01 treatment, the cells were treated with 1 μg/mL lipopolysaccharide (LPS) and BDX-01 (1 × 10^7^ CFU/mL) for 4 h. For LPS treatment, 1 μg/mL LPS was subjected to cells for 4 h. For the separation of different components of BDX-01, the intact bacterial suspension was directly preserved, and then part of the BDX-01 culture suspension was centrifuged at 9,000 rpm for 10 min to separate into bacterial precipitate and culture supernatant. The bacterial precipitate was heat inactivated at 100°C for 10 min, and then the BDX-01 culture supernatant (BDS) was filtered through a cellulose acetate filter with a pore size of 0.2 μm. The Caco-2 cells were pretreated with live BDX-01 (MOI = 1:100), heat-killed BDX-01 (MOI = 1:100), and BDS (50%) for 4 h and TNF-α (100 ng/mL) for another 4 h. After incubation, cells were collected for qRT-PCR.

### 2.5 Animal study

The experimental protocol was approved by the Institutional Animal Care and Use Committee of Southern Medical University (approval number L2018225), and all *in vivo* experiments were performed under the guidelines of our institution for the use of laboratory animals. All SPF male C57BL/6J mice aged 6–8 weeks and 20–25 g in body weight were purchased from the Animal Center of the Southern Medical University, Guangzhou, China. To assess *in vivo* BDX-01 safety, mice were assigned randomly into two groups (n = 5–8/group). Each group was given either 5 × 10^11^ CFU/d BDX-01 in phosphate-buffered saline (PBS, Solarbio Life Sciences, China) or 0.5 mL/d PBS by oral gavage for 5 days. General condition and body weight were monitored daily for 14 days after intragastric administration. At the end of the experiment, mice were sacrificed and blood samples were collected for hematological and serum biochemical analysis. The mice were anesthetized with 100 mg/kg pentobarbital sodium (Nanfang Hospital, Southern Medical University) and sacrificed by cervical dislocation. The stomach, colon, liver and spleen were weighed and subjected to histopathological examination.

For the acute colitis experiment, colitis induction was performed as previously described with slight modifications (Chassaing et al., 2014). Briefly, 2.5% (w/v) dextran sulfate sodium (DSS; MW = 36,000–50,000 Da; Macklin Biochemical Co., Ltd., Shanghai, China) was added to the drinking water of mice and administered continuously for 8 days. Changes in disease activity index (DAI) were assessed as previously described (Jang et al., 2019) according to the following criteria: (1) weight loss (%), (2) stool consistency, and (3) fecal blood (Supplementary Table S1).

The treatment groups were normal (Control), DSS model (Model), mesalazine (Mesalazine), and *B. dorei* (BDX-01), with eight mice randomly assigned per group. During the 7-day pre-intervention, mice in the BDX-01 group were administered by gavage a viable overnight culture of 1 × 10^9^ CFU/kg BW/d BDX-01. Simultaneously, the Mesalazine and Model groups were administered equivalent volumes of the culture medium by gavage. Except for those in the Control group, mice in each group received 2.5% (w/v) DSS in drinking water for 8 days. Starting 3 days before DSS intervention, the Mesalazine group was administered 200 mg/kg BW/d mesalazine (Macklin Biochemical Co., Ltd.) dissolved in PBS. Body weight and mental conditions were monitored throughout the experiment. The stool samples were collected on the day the experiment ended. On day 8 after DSS treatment, the mice were euthanized, and their colons and feces were collected.

### 2.6 Antibiotic treatment

Mouse gut microbiota were depleted as previously described with minor modifications (Ke et al., 2021). Briefly, for 4 days before the BDX-01/culture medium treatment and the colitis induction, the mice were administered by gavage an antibiotic cocktail (Abx) comprising 100 mg/kg/d vancomycin, 200 mg/kg/d metronidazole, 40 mg/kg/d gentamicin, and 200 mg/kg/d ampicillin. Feces were collected on day 5 and subjected to gel electrophoresis to assess the effects of antibiotics on depleting gut microbiota. Then, the Abx, Abx + DSS, and Abx + DSS + BDX-01 groups were designated, and eight mice were randomly assigned to each. The interventions used were consistent with those administered to the corresponding colitis groups described above.

### 2.7 Histological analysis

The tissues were fixed with 4% paraformaldehyde and embedded in paraffin, cut into 4-μm sections, and stained with hematoxylin-eosin (HE) for pathological analysis. The histological scores were classified into one of the following groups: (1) loss of epithelium, (2) crypt damage, (3) depletion of goblet cells, or (4) infiltration of inflammatory cells (Supplementary Table S2) (Jang et al., 2019).

### 2.8 RNA extraction and qRT-PCR

Total RNA was extracted with TRIzol reagent (Invitrogen, Carlsbad, CA, United States) according to the operating instructions. The mRNA was transformed into cDNA with reverse transcriptases (TaKaRa Bio Inc., Kusatsu, Shiga, Japan). Quantitative real-time PCR was performed using SYBR Premix Ex Taq (Takara, Japan) in a Roche Light Cycler 96 Real-time System (Roche Diagnostics, Basel, Switzerland). Each sample was run in triplicate. Each sample was detected in triplicate 10-μL reaction volumes and assessed using the comparative cycle (2^−ΔΔCT^) method (Livak and Schmittgen, 2001). The results were normalized to the expression of GADPH and calculated relative to the control group. The primers used are listed in Supplementary Table S3.

### 2.9 Analysis by western blot

Colon samples were collected and homogenized with a tissue breaker in 1× radioimmunoprecipitation assay (RIPA, Solarbio Life Sciences, Beijing, China) buffer with phenylmethylsulfonyl fluoride (PMSF, Solarbio Life Sciences, Beijing, China; 1:1,000). The mixtures were centrifuged at 15,000 × g and 4°C for 10 min. The supernatant was collected and the protein concentration was determined using a bicinchoninic acid (BCA) protein assay kit (Solarbio Life Sciences, Beijing, China). Proteins were separated by 12% (w/v) SDS-polyacrylamide gel and transferred to polyvinylidene difluoride (PVDF) membranes (Bio-Rad Laboratories, Hercules, CA, United States). The membranes were blocked for 1 h with 5% (v/v) skimmed milk and incubated at 4°C overnight with rabbit anti-caspase-1 monoclonal antibody (1:1,000 dilution; Cell Signaling Technology (CST), Danvers, MA, United States), rabbit anti-NLRP3 monoclonal antibody (1:1,000 dilution; Proteintech Group, Rosemont, IL, United States), rabbit anti-IL-1β monoclonal antibody (1: 1,000 dilution; Proteintech Group), rabbit anti-GSDMD monoclonal antibody (1:1,000 dilution; CST), rabbit anti-FXR monoclonal antibody (1: 1,000 dilution; Proteintech Group), rabbit anti-G-protein bile acid receptor-1 (TGR5) monoclonal antibody (1: 1,000 dilution; Proteintech Group), rabbit anti-vitamin D receptor (VDR, NR1H1) monoclonal antibody (1: 1,000 dilution; Proteintech Group), rabbit anti-pregnane-X-receptors (PXR, NR1H2) monoclonal antibody (1: 1,000 dilution; Proteintech Group), rabbit anti-FGF15 monoclonal antibody (1: 1,000 dilution; Proteintech Group) and rabbit anti-β-actin monoclonal antibody (1:10,000 dilution; ABclonal, Woburn, MA, United States). The membranes were then incubated with horseradish peroxidase (HRP)-labeled secondary antibodies at 25°C for 1 h. Protein bands were visualized with an enhanced chemiluminescence (ECL) kit (Fdbio Science, Hangzhou, Zhejiang, China). Band intensities were normalized relative to β-actin.

### 2.10 DNA extraction and PCR amplification

Bacteria genomic DNA was extracted from mice’ feces with a TIANGEN stool DNA kit (No. DP328; TIANGEN Biotech Co., Ltd., Beijing, China) according to operating instructions and diluted to 1 ng/μL. Fecal DNA was amplified with V4 primers 514F (5′-GTGCCAGCMGCCGCGGTAA- 3′) and 805R (5′-GGACTACHVGGGTWTCTAAT-3′) and a TIANGEN 2 × Taq PCR MasterMix (No. KT201; TIANGEN Biotech Co., Ltd.) according to operating instructions (Yin et al., 2015).

### 2.11 16S rRNA sequencing of microbial DNA and microbial community analysis

Total genomic DNA was extracted from the mice’s stool using the TIANamp Stool DNA Kit (Tiangen, Beijing, China). The V3–V4 region of the 16S rRNA gene was amplified using 338F and 806R primers and sequenced using an Illumina HiSeq 2,500 platform (Illumina, San Diego, CA, United States) by Majorbio BioPharm Technology Co., Ltd. (Shanghai, China). Paired end reads were merged via the rapid length adjustment of short reads, followed by sequence analysis using UPARSE (Edgar, 2013). Subsequently, sequences with ≥97% similarity were subsequently assigned to the same operational taxonomic units (OTUs) (Magoč and Salzberg, 2011). Then, representative sequences were selected using the QIIME software from each OTU, which were aligned and annotated based on the SILVA database (v138) (Caporaso et al., 2010).

### 2.12 Fecal BSH activity

BSH activity was determined by measuring the amount of amino acids released from conjugated bile salts, as previously described with minor modifications (Kumar et al., 2012). BSH activity of BDX-01 was measured *in vitro*, and *Lactobacillus* plantarum strain lp91 was used as a positive control. Fecal bacterial BSH activity was assayed according to CA production from fecal TCA. Briefly, protein extracts were prepared by sonicating 0.5 g of stool samples in 1 mL of PBS. The samples were centrifuged at 12,000 × g for 10 min and 1 mL of supernatant was collected and incubated with 1.8 mL of PBS and 0.1 mL of 0.1 M trichloroacetic acid (Shanghai Macklin Biochemical Co., Ltd., Shanghai, China) at 37°C for 30 min. Reactions were stopped by adding with 0.1 mL of trichloroacetic acid for 1 min. The mixtures were centrifuged and 1 mL of supernatant collected was mixed with 1 mL of 2 M trichloroacetic acid buffer and 1 mL of ninhydrin reagent [0.5 mL of 1% (w/v) ninhydrin in 0.5 M citrate buffer (pH 5.5), 1.2 mL of 30% (v/v) glycerol, and 0.2 mL of 0.5 M citrate buffer (pH 5.5)]. The mixture was vortexed to mix well, boiled for 15 min, and then cooled on ice. The taurine standard was then read for absorbance at 570 nm. Unit activity of BSH was expressed as the amount of enzyme that releases 1 mmol amino acid (AA)/min from the substrate.

### 2.13 Bile acid analysis

Stock solutions were prepared by diluting the standard to 10 mM. Calibration standard solutions were prepared by stepwise dilution of the standard containing isotopically labeled internal standard mixture identical in concentration to the samples. Each stool sample (25 mg) was weighed and transferred to the Eppendorf tube (Eppendorf GmbH, Hamburg, Germany). Then 1,000 μL extract solution (2:2:1 acetonitrile-methanol-water with 0.1% (v/v) formic acid and isotopically labeled internal standard mixture) was added, and the samples were vortexed, sonicated in an ice bath, incubated, and centrifuged. The supernatants were transferred to liquid chromatography-mass spectrometry (LC-MS) vials for ultrahigh-performance liquid chromatography-tandem mass spectrometry (UHPLC-MS/MS) analysis by BIOTREE (Shanghai, China).

### 2.14 Statistical analysis

All data are displayed as mean ± standard deviation (SD). Data were analyzed using an unpaired *t*-test, Kruskal–Wallis test, Mann–Whitney *U* test, or one-way analysis of variance (ANOVA). A *p*-value of < 0.05 was considered statistically significant. Statistical analyses were performed using Prism v. 9.0.0 (GraphPad Software, Inc., La Jolla, CA, United States) and SPSS v. 19.0 for Windows (SPSS Inc., Chicago, IL, United States).

## 3 Results

### 3.1 Morphological characteristics and sequence identification of *B. dorei* BDX-01

**FIGURE 1:**
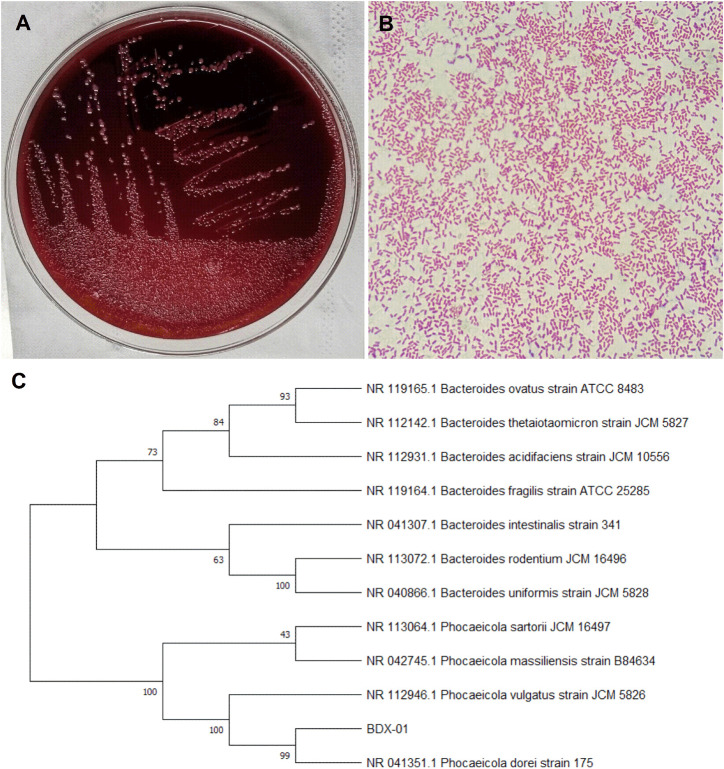
Morphological characteristics and sequence identification of BDX-01. **(A)** BDX-01 colonies on BHI (3% sheep blood) agar after anaerobic culture at 37°C for 48 h. **(B)** Gram staining under an optical microscope (×1,000). **(C)** Sequence identification and phylogenetic tree.

The bacterial strain *B. dorei* BDX-01 (BDX-01) was isolated from the stool of a healthy human and morphologically resembled *B. dorei*. Gram staining and direct microscopic observation revealed that this strain was Gram-negative and had a short rod shape with rounded ends. The 16S rRNA gene sequence analysis demonstrated that the strain shared 99% nucleotide sequence similarity with the *B. dorei* 175 strain. The complete genome was 5,521,745 bp in length and contained on a single chromosome, with an average GC content of 42.01%. BDX-01 and *B. dorei* 175 strain had an ANI of 99.97%. Hence, the isolate was a novel *B. dorei* strain (Figures 1A–C).

### 3.2 Safety assessment of BDX-01

Before the colitis study, we assessed the virulence factor, antibiotic resistance, cytotoxicity, and animal toxicity of BDX-01 to confirm its *in vitro* and *in vivo* safety. According to the blast analysis of the VFDB database, 14 virulence factor homologs were identified in BDX-01 genome (Table 1), most of which correlate with cellular structure or physiological activities, and none have been reported as the pathogenesis of *B. dorei*. The antibiotic resistance genes identified in the genome of BDX-01 are summarized in Table 2. All drug-resistance genes were located on the chromosome rather than on plasmids. Based on the clinical resistance classification, its MIC was >32 μg/mL (D’Aimmo et al., 2007). It was sensitive to cefoperazone, meropenem, tetracycline, and ciprofloxacin and particularly sensitive to clindamycin and clarithromycin. The latter two antibiotics inhibited protein synthesis in BDX-01 (MIC = 0.125 μg/mL and 0.5 μg/mL, respectively) (Table 3).

**TABLE 1:** Putative virulence-associated genes identified in the genome of *B. dorei* BDX-01.

Gene ID	VFDB_ID	Name	Function
GE001325	gmd	VF0367	GDP-mannose 4,6-dehydratase
GE004165	gmd	VF0367	GDP-mannose 4,6-dehydratase
GE002405	acpXL	CVF383	acyl carrier protein
GE000706	htpB	VF0159	Hsp60, 60K heat shock protein HtpB
GE002085	ddhA	VF0392	glucose-1-phosphate cytidylyltransferase
GE002423	glf	VF0323	UDP-galactopyranose mutase
GE001345	ddhA	VF0392	glucose-1-phosphate cytidylyltransferase
GE002948	mip	VF0153	macrophage infectivity potentiator Mip
GE000630	clpP	VF0074	ATP-dependent Clp protease proteolytic subunit
GE001758	kdsA	CVF383	2-dehydro-3-deoxyphosphooctonate aldolase
GE001923	AHA_1389	CVF786	CobQ/CobB/MinD/ParA family protein
GE003805	cap8J	VF0003	capsular polysaccharide synthesis enzyme Cap8J
GE003888	rffG	CVF494	dTDP-glucose 46-dehydratase
GE001801	sodB	VF0169	superoxide dismutase

**TABLE 2:** Putative antibiotic resistance genes identified in the genome of *B. dorei* BDX-01.

Gene ID	Name	Drug
GE003808	ErmG	macrolide; lincosamide; streptogramin antibiotic
GE000738	adeF	tetracycline; fluoroquinolone
GE000859	adeJ	rifamycin; penem; macrolide; carbapenem; cephalosporin; diaminopyrimidine; tetracycline; fluoroquinolone; lincosamide; phenicol
GE002282	tetQ	tetracycline
GE001552	adeF	tetracycline; fluoroquinolone

**TABLE 3:** Relative antibiotic resistance profiles of *B. dorei* BDX-01 and *B. dorei* 175 (μg/mL).

Antibiotics	*B. dorei* 175	*B. dorei* BDX-01
Ampicillin	MIC ≥ 512	MIC ≥ 512
Vancomycin	MIC ≥ 256	MIC ≤ 128
Cefoperazone	MIC ≤ 16	MIC ≤ 8
Meropenem	MIC ≤ 32	MIC ≤ 32
Clindamycin	MIC ≤ 0.125	MIC ≤ 0.125
Chloromycetin	MIC ≤ 64	MIC ≥ 64
Clarithromycin	MIC ≤ 0.5	MIC ≤ 0.5
Gentamicin	MIC ≥ 512	MIC ≥ 512
Streptomycin	MIC ≥ 512	MIC ≥ 512
Tetracycline	MIC ≤ 8	MIC ≤ 8
Metronidazole	MIC ≥ 512	MIC ≥ 512
Ciprofloxacin	MIC ≤ 32	MIC ≤ 32
Trimethoprim	MIC ≤ 512	MIC ≤ 512
Polymyxin B	MIC ≥ 512	MIC ≥ 512

**FIGURE 2:**
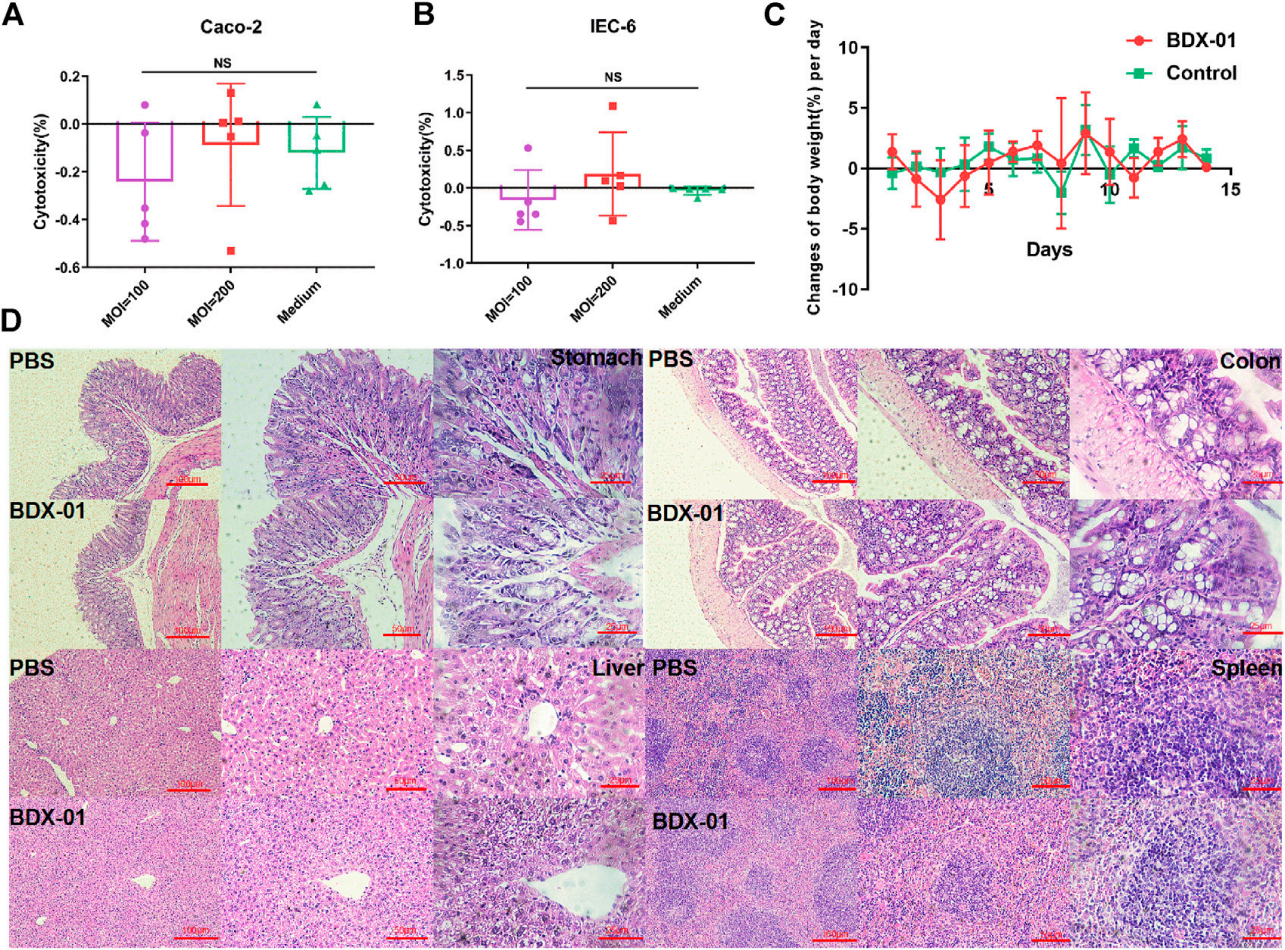
BDX-01 is non-pathogenic to Caco-2 and IEC-6 cells and C57BL/6J mice. **(A,B)** BDX-01 cytotoxicity to Caco-2 **(A)** and IEC-6 **(B)** cells at 100:1 and 200:1 infection ratios for 4 h. The culture medium was the negative control (*n* = 5). **(C)** Changes in the BW (%)/d in the treatment and control groups. SPF C57BL/6J normal mice (*n* = 8) were treated with 5 × 10^11^ CFU/d BDX-01 for 5 days and observed for 14 days. The Control group was treated with PBS. **(D)** Photomicrographs of stomachs, colons, livers, and spleens of mice in the control (upper) and treatment (lower) groups. No significant lesions were observed.

Cytotoxicity assays using IEC-6 and Caco-2 cells showed no significant difference between the treatment and control groups (*p >* 0.05). Therefore, intact viable BDX-01 cells were not cytotoxic *in vitro* (Figures 2A, B). In the acute toxicity experiments, neither death nor treatment-related toxicity occurred in mice. Body weight did not differ significantly between the groups (Figure 2C). No histopathological injury to the stomach, colon, liver, and spleen was found in both groups (Figure 2D). In addition, hematologic analysis revealed no significant differences in blood routine indices and hepatic and renal function between the groups (Supplementary Table S4).

### 3.3 BDX-01 administration alleviated colitis and mucosal injury

**FIGURE 3:**
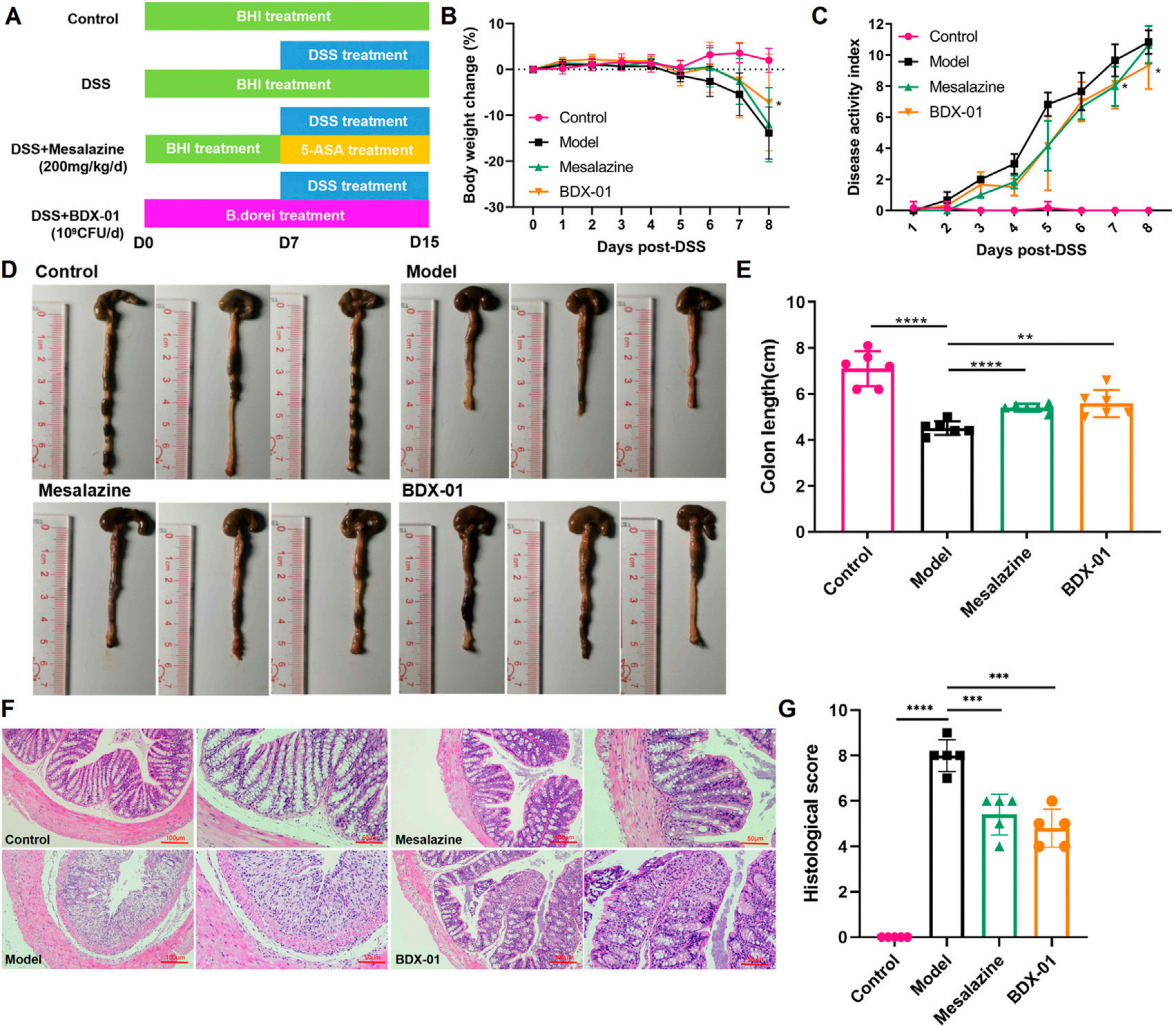
BDX-01 supplementation alleviated mouse DSS-induced colitis. **(A)** Protocol for BDX-01 supplement to mouse DSS-induced colitis model. The DSS, Mesalazine, and BDX-01 groups were administered 100 μL BHI medium or 100 μL BHI medium plus 100 μL BDX-01 culture containing 1 × 10^9^ CFU/mL, respectively, through an intragastric cannula for 15 days. All mice received 2.5% DSS in drinking water between days 7 and 15. **(B)** % BW change during DSS treatment relative to baseline (day 0). **(C)** DAI scores during DSS treatment. **(D)** Representative colon morphology and length. **(E)** Comparison of colon lengths among the four groups. **(F)** Representative HE-stained histological sections (100× and 200×). **(G)** Comparison of colonic histopathological scores among the four groups. Statistical analysis was performed in comparison with DSS group mice. Data represent the means ± SD (*n* = 6–8). *p < 0.05; **p < 0.01; ***p < 0.001; ****p < 0.0001 (unpaired *t*-test, Kruskal–Wallis test, and one-way ANOVA, followed by *post hoc* test).

We investigated the effects of BDX-01 on a mouse DSS-induced colitis model (Figure 3A). DSS administration significantly reduced body weight, shortened colon length, and increased the DAI score in mice. Weight loss (Figures 3B, C) and colon length reduction (Figures 3D, E) were significantly reduced in the Mesalazine and BDX-01 groups compared with the Model group. Furthermore, BDX-01 treatment markedly reduced the DAI score of mice (Figures 3D, E). Hence, BDX-01 treatment improved the symptoms of DSS-induced acute colitis.

HE staining revealed inflammatory cell infiltration, crypt loss, and mucosal injury in the colon tissue of the Model group (Figure 3F). In contrast, the Mesalazine and BDX-01 groups displayed nearly complete colon tissue integrity. The histopathological scores of the Model group (8.00 ± 0.71) were significantly increased and significantly decreased in the Mesalazine (5.40 ± 0.89) and BDX-01 (4.80 ± 0.84) groups (Figure 3G). Therefore, BDX-01 treatment alleviated DSS-induced colon histopathological damage.

### 3.4 Treatment with BDX-01 mitigated DSS-induced gut microbiota dysbiosis

**FIGURE 4:**
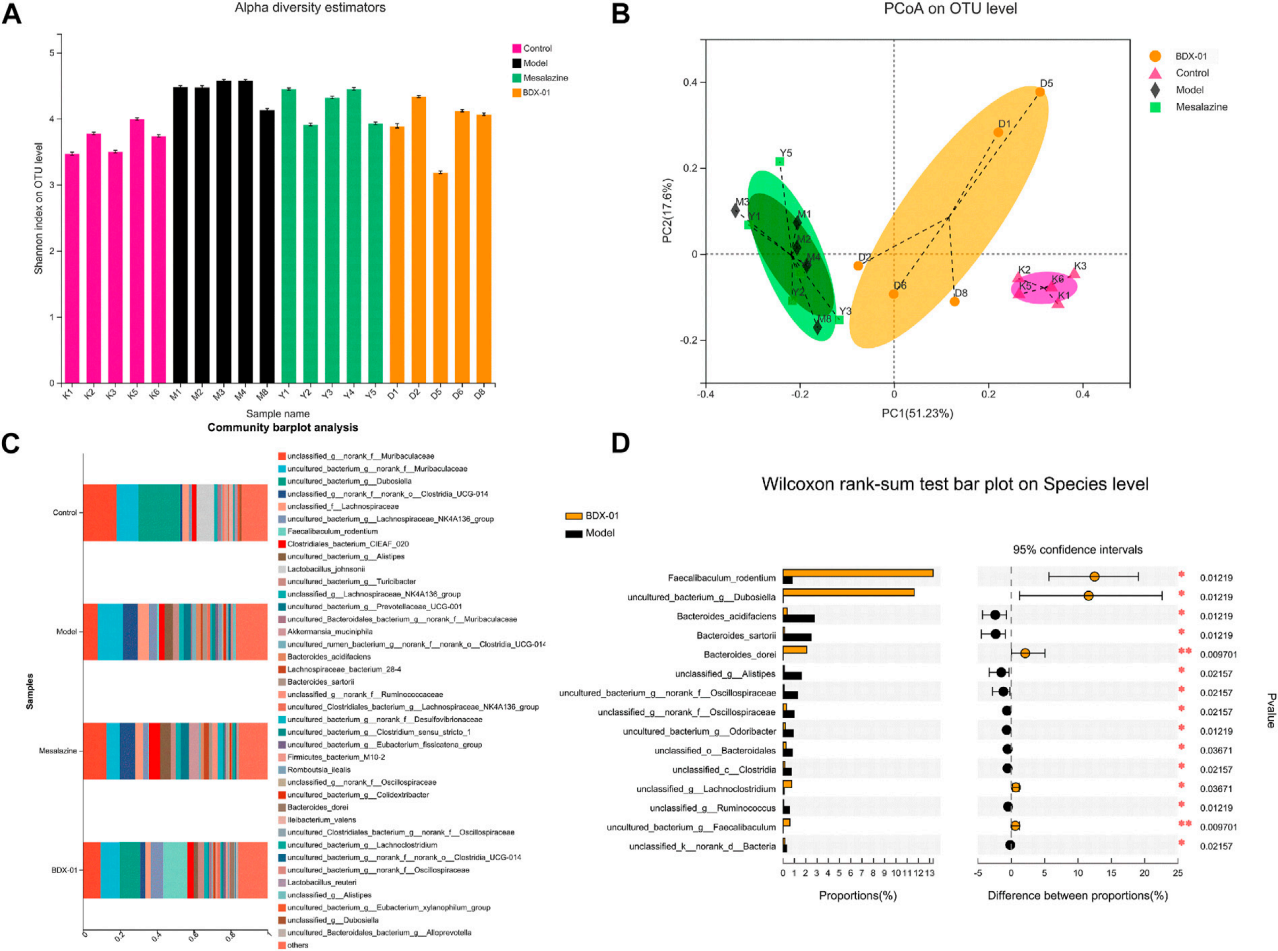
BDX-01 mitigated DSS-induced changes in gut microbiota composition. **(A)** α-diversity evaluated based on the Shannon index. **(B)** β-diversity assessed using PCoA based on weighted UniFrac distances. **(C)** Average relative species-level abundances of the gut microbiota of the four mouse groups. **(D)** Differential species-level abundances of bacterial OTUs in the BDX-01 and DSS groups. Only differentially abundant species are shown. Data represent the means ± SD (*n* = 5). *p < 0.05; **p < 0.01; ***p < 0.001; ****p < 0.001 (one-way ANOVA, followed by *post hoc* test).

Dysbiosis of the intestinal microbiota is characteristic of UC. Therefore, we investigated whether BDX-01 modulates the composition of the gut microbiota and confers protection against UC. Alpha-diversity was not significantly different among the four groups based on their Shannon indices (Figure 4A). The beta-diversity was evaluated based on a principal coordinate analysis (PCoA) of the weighted UniFrac distance. The gut microbiota of DSS-treated mice differed significantly from that of control mice. However, the administration of BDX-01 ameliorated the shift in the gut microbiota induced by DSS challenge. In contrast, mesalazine had no apparent significant effect on gut dysbiosis (Figure 4B).

We then used a Wilcoxon rank test to compare the four groups in terms of their bacterial distributions at various taxonomic levels. The relative abundances of Firmicutes and Actinobacteriota were significantly lower, and that of Desulfobacterota was significantly higher in the Model and Mesalazine groups than in the Control group. The abundances of *Verrucomicrobiota* was significantly higher in the Mesalazine group. The abundances of Firmicutes and Actinobacteriota were significantly higher in the BDX-01 group than in the other groups (Supplementary Figure S2).

In the family-level (Supplementary Figure S3), the abundances of Erysipelotrichaceae, Lactobacillaceae, and Akkermansiaceae were lower, and those of Bacteroidaceae, Rikenellaceae, Oscillospiraceae, Prevotellaceae, Ruminococcaceae, and Desulfovibrionaceae were higher in the Model and Mesalazine groups than in the Control group. Erysipelotrichaceae in the BDX-01 group and *Akkermansiaceae* in the Mesalazine group were markedly higher than those in the Model group.

In the genus-level, the relative abundances of *Dubosiella* and *Lactobacillus* were significantly lower in the Model group than in the Control group. However, mesalazine treatment significantly increased the relative abundance of *Akkermansia* and *Lachnoclostridium*. Supplementation with BDX-01 notably increased the relative abundances of *Dubosiella* and *Faecalibaculum* (Supplementary Figure S4). *Dubosiella*, *Faecalibaculum_rodentium*, and *Bacteroides_dorei* were higher in the BDX-01 group than in the Model group (Figures 4C, D). Thus, BDX-01 alleviates colitis and regulates gut microbiota composition.

### 3.5 BDX-01 affected bile acid metabolism and activated FXR in the colons of mice with DSS-induced colitis

**FIGURE 5:**
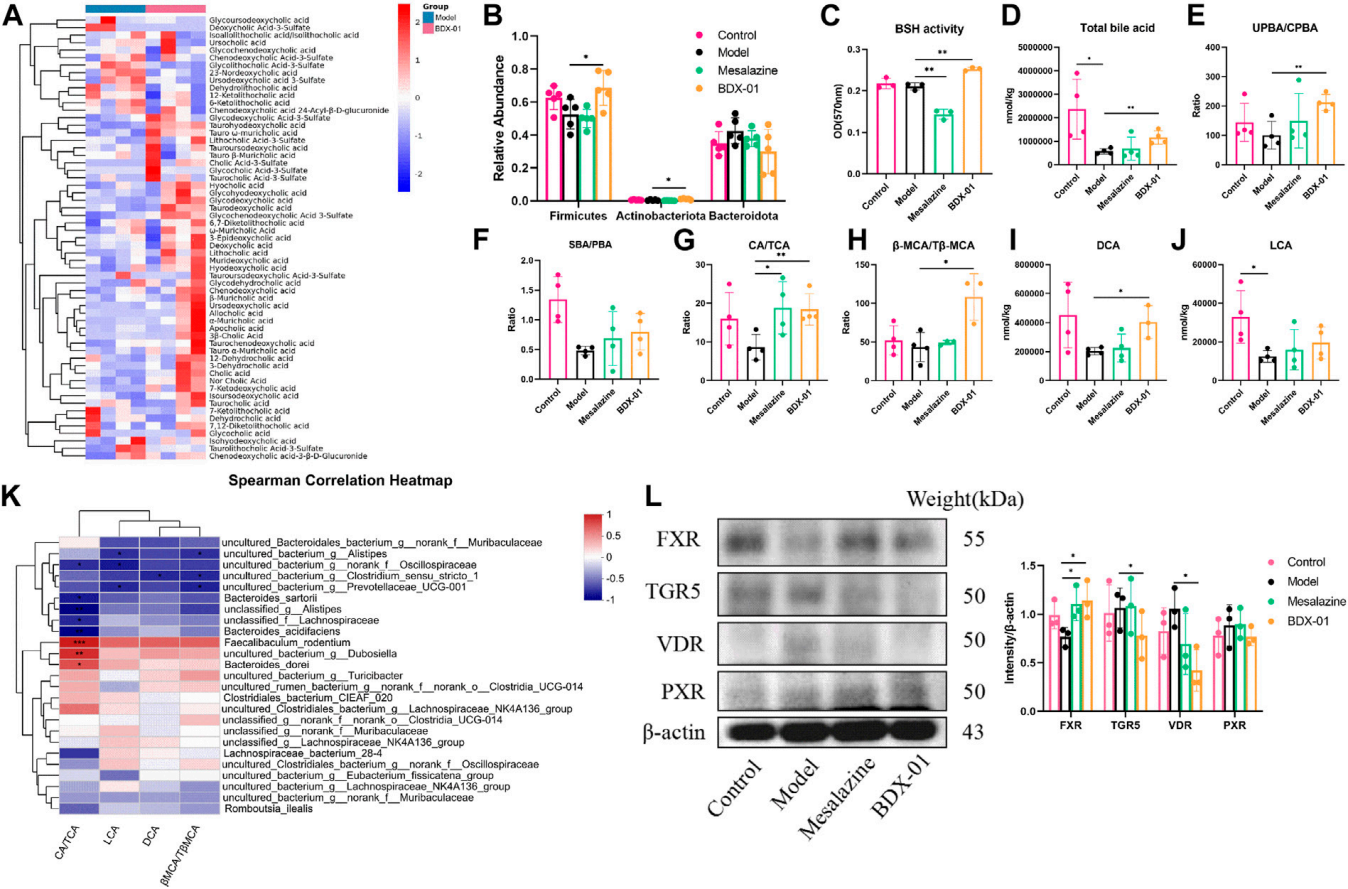
Treatment with BDX-01 altered the gut microbiota and BA profile and activated FXR. **(A)** Fecal BA profiles of the DSS and BDX-01 groups. **(B)** Fecal microbiota that exhibit a change in BSH activity. **(C)** Fecal BSH activity. **(D)** Total BA levels. **(E)** UPBA/CPBA ratio. **(F)** SBA/PBA ratio. **(G)** CA/TCA ratio. **(H)** βMCA/T-βMCA ratio. **(I)** DCA level. **(J)** LCA level. **(K)** Heatmap of correlations among predominant bacterial taxa and fecal BA in the DSS and BDX-01 groups. **(L)** The expression of different colonic bile acid receptors were determined using western blotting analysis. Data represent the means ± SD (*n* = 3–6). *p < 0.05; **p < 0.01; ***p < 0.001; ****p < 0.0001. OD, optical density.

Changes in BA composition and metabolism have been linked to UC. A fecal bile acid analysis revealed that BDX-01 treatment significantly altered the fecal BA profile (Figure 5A). Enzyme activity assays in patients with IBD showed impaired BA deconjugation due to decreased BSH activity (Duboc et al., 2013). BDX-01 exhibited high BSH activity *in vitro* (Supplementary Figure S5). Gut microbiota analyses revealed that Firmicutes, Actinobacteria, and Erysipelotrichaceae have high BSH activity (Jones et al., 2008; Labbé et al., 2014). These taxa were significantly increased in response to the BDX-01 treatment (Figure 5B). An assay of fecal samples confirmed that BDX-01 significantly increased intestinal BSH activity (Figure 5C). Compared to the Control group, the Model group presented significantly decreased total fecal BA level and unconjugated primary bile acid (UPBA):conjugated primary bile acid (CPBA) and SBA:PBA ratios. Therefore, intestinal BA excretion, deconjugation, and 7α-dehydroxylation were altered in the Model group (Figures 5D–F). The CA/TCA and β-MCA/Tβ-MCA ratios and DCA level were significantly reduced in the Model group but could be reversed by BDX-01 treatment (Figures 5G–J). Spearman rank correlation analysis (Figure 5K) showed significant positive correlations between fecal CA/TCA and the relative abundances of *Faecalibaculum_rodentium, Dubosiella*, and *Bacteroides_dorei*. βMCA/TβMCA, DCA, and LCA were positively correlated with the aforementioned bacteria. In contrast, BAs were negatively correlated with the abundances of *Bacteroides_acidifaciens*, *Bacteroides_sartorii,* and *Alistipes* in the Model group.

Elevated fecal CA/TCA and β-MCA/Tβ-MCA ratios and DCA in response to BDX-01 treatment may regulate host metabolism and immunity by acting on different bile acid receptors. The various bile acids activate several nuclear receptors in the gut: the TGR5 that is activated by LCA and DCA; the PXR that are activated by CDCA, LCA, and DCA; and the VDR that is activated by LCA and DCA, in addition to vitamin D (Fiorucci et al., 2022). Western blotting analysis confirmed that BDX-01 only upregulated FXR among the receptors (Figure 5L; Supplementary Figure S6). These results suggest that BDX-01 may alleviate colitis by regulating the bile acid-FXR pathway. Detailed relative BA alterations in feces are shown in Supplementary Table S5.

### 3.6 BDX-01 regulated the production of proinflammatory cytokines and suppressed the NLRP3 inflammasome signaling pathway

**FIGURE 6:**
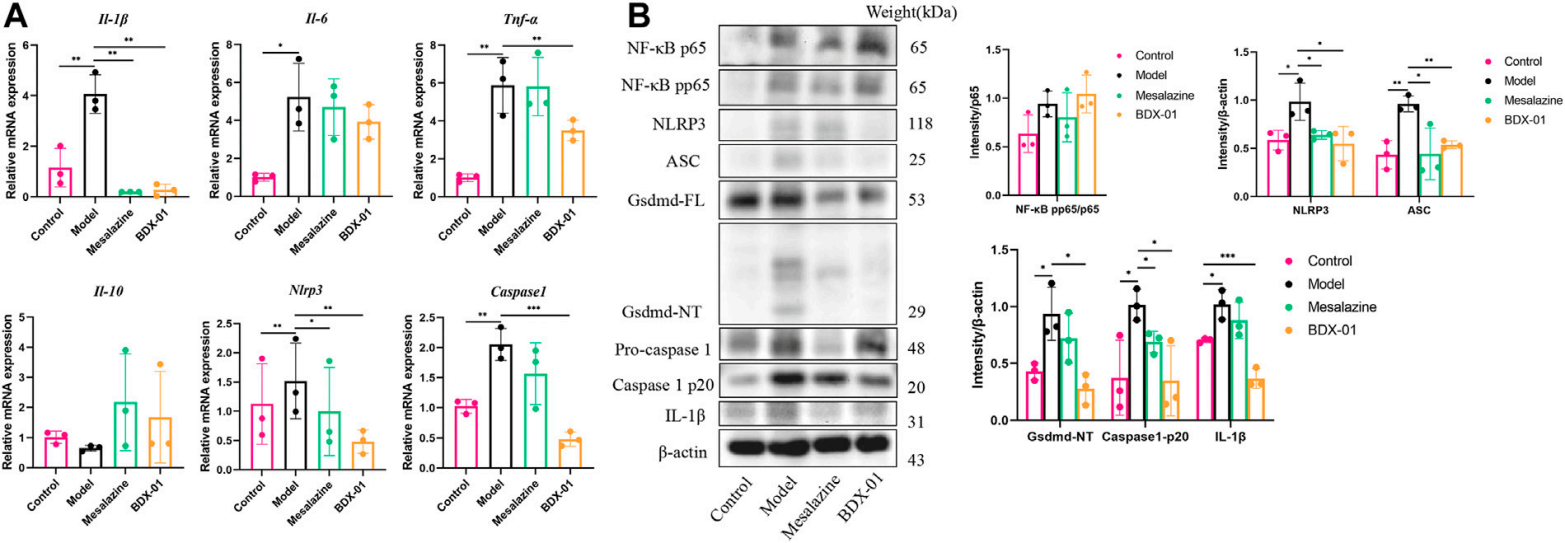
BDX-01 regulated proinflammatory cytokine production and suppressed the NLRP3 inflammasome signaling pathway. **(A)** mRNA levels of *Il-1β*, *Tnf-α*, *Il-6*, *Il-10*, *Nlrp3*, and *Caspase-1* in colon tissues were determined using qRT-PCR. **(B)** NF-κB, NLRP3, ASC, caspase-1, gsdmd, and IL-1β expression levels in the DSS group were determined using western blotting analysis. Data represent the means ± SD (*n* = 3). *p < 0.05; **p < 0.01; ***p < 0.001; ****p < 0.0001 (unpaired *t*-test, Kruskal–Wallis test, and one-way ANOVA, followed by *post hoc* test).

We examined the proinflammatory cytokines of the colon tissue to evaluate the effects of BDX-01 on the inflammatory response. DSS administration significantly upregulated the proinflammatory cytokines TNF-α, IL-1β, and IL-6 compared with the control. In contrast, they were markedly downregulated in the Mesalazine and BDX-01 groups (Figure 6A). The expression of the anti-inflammatory cytokine IL-10 was downregulated and relatively upregulated in the DSS and BDX-01 groups, respectively (Figure 6A).

The FXR agonism exert anti-inflammatory effects by inhibiting inflammatory signaling pathways, including the NF-κB signaling pathway and the NLRP3 inflammasome signaling pathway (Gadaleta et al., 2011; Hao et al., 2017). The mRNA and/or the protein expression levels of NF-κB pp65, NLRP3, ASC, caspase-1, gsdmd, and IL-1β were significantly increased in the Model group colon tissues. Administration of BDX-01 and mesalazine markedly reversed the overexpression of the NLRP3 inflammasome signaling pathway but did not inhibit NF-κB activation (Figures 6A, B; Supplementary Figure S7). Thus, BDX-01 could modulate the NLRP3 inflammasome signaling pathway and alleviate inflammation.

### 3.7 BDX-01 ameliorated DSS-induced colitis by directly activating FXR and inhibiting the NLRP3 pathway

**FIGURE 7:**
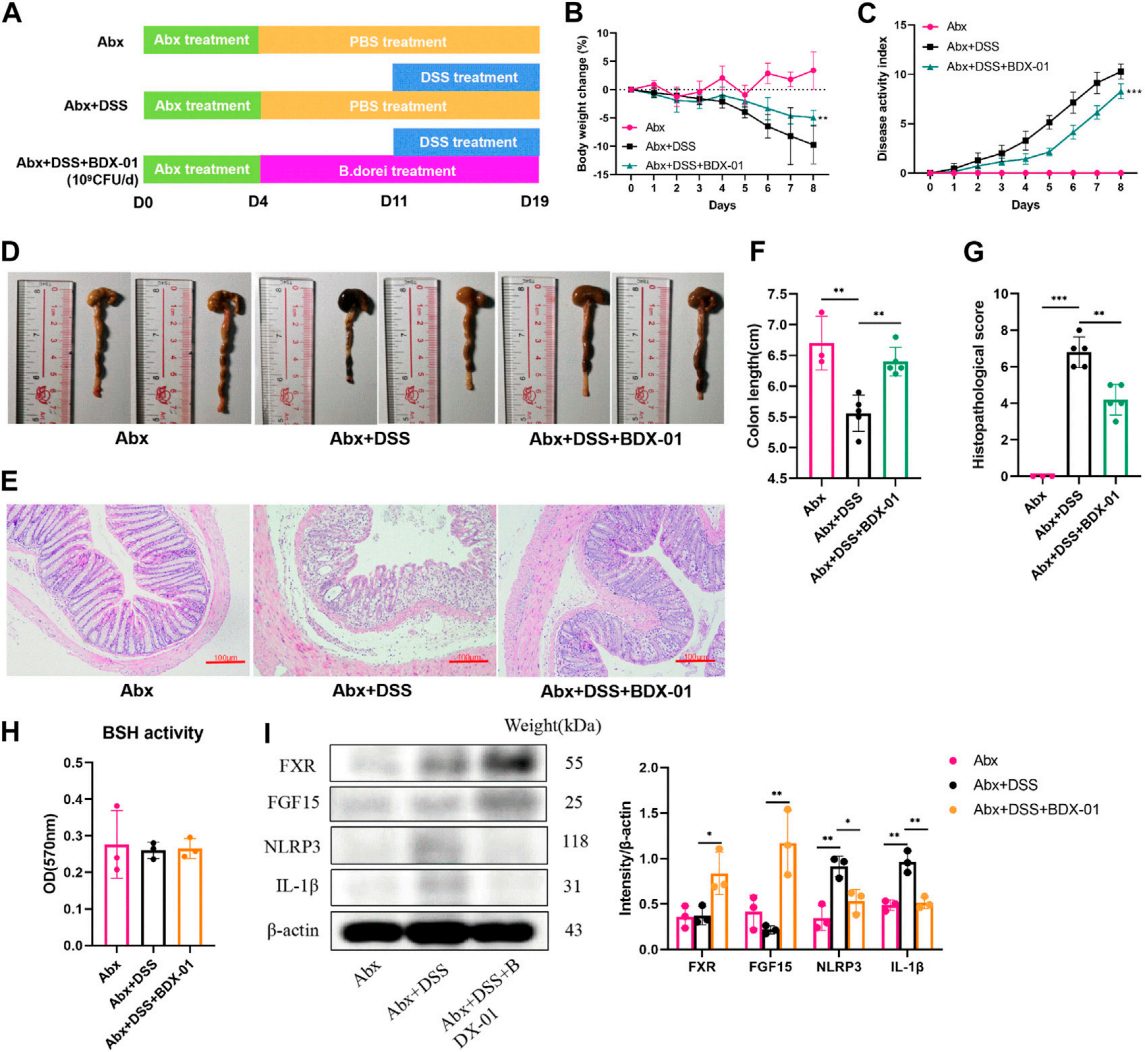
BDX-01 supplementation alleviated mouse DSS-induced colitis following antibiotic treatment. **(A)** Mice were administered antibiotics for 4 days to deplete the gut microbiota before DSS treatment. The Abx + DSS and Abx + BDX-01 + DSS groups were administered 100 μL culture medium and 100 μL of 1 × 10^9^ CFU culture of BDX-01 by gavage for 15 days, respectively. **(B)** Body weight change, **(C)** disease activity index score, **(D)** representative images of colon and **(E)** colon length, **(F)** representative microscopic images of HE-stained sections (100×), and **(G)** histopathology score. **(H)** Fecal BSH activity. **(I)** FXR, FGF15, NLRP3, and IL-1β expression determined using western blotting analysis. Data are mean ± SD (*n* = 3–5). *p < 0.05; **p < 0.01; ***p < 0.001; ****p < 0.0001 (unpaired *t*-tests, Kruskal–Wallis test, and one-way ANOVA, followed by *post hoc* test).

Mice were given an oral antibiotic (Abx) to deplete the gut microbiota and determine their roles in alleviating colitis with BDX-01 (Figure 7A). All microbiota were effectively depleted after oral antibiotic cocktail administration for 4 days (Supplementary Figure S1). As expected, mice in the Abx + DSS group showed symptoms of colitis (weight loss and elevated DAI score) and colonic histopathological damage compared with those in the Abx group. Body weight loss (Figure 7B), DAI score (Figure 7C), colon length (Figures 7D, E), and histopathology score (Figures 7F, 7G) were markedly decreased in the Abx + DSS + BDX-01 group compared with the Abx + DSS group. Fecal BSH activity did not differ significantly among the three groups (Figure 7H). However, treatment with BDX-01 upregulated FXR and its downstream factor FGF15 and downregulated NLRP3 and IL-1β compared with the Abx + DSS group (Figure 7I; Supplementary Figure S8). These data indicate that, in addition to regulating the gut microbiota, BDX-01 mitigates DSS-induced colitis by directly activating the FXR-NLRP3 pathway.

### 3.8 BDX-01 inhibits the NLRP3 inflammasome signaling pathway in intestinal cells and macrophages by activating FXR

**FIGURE 8:**
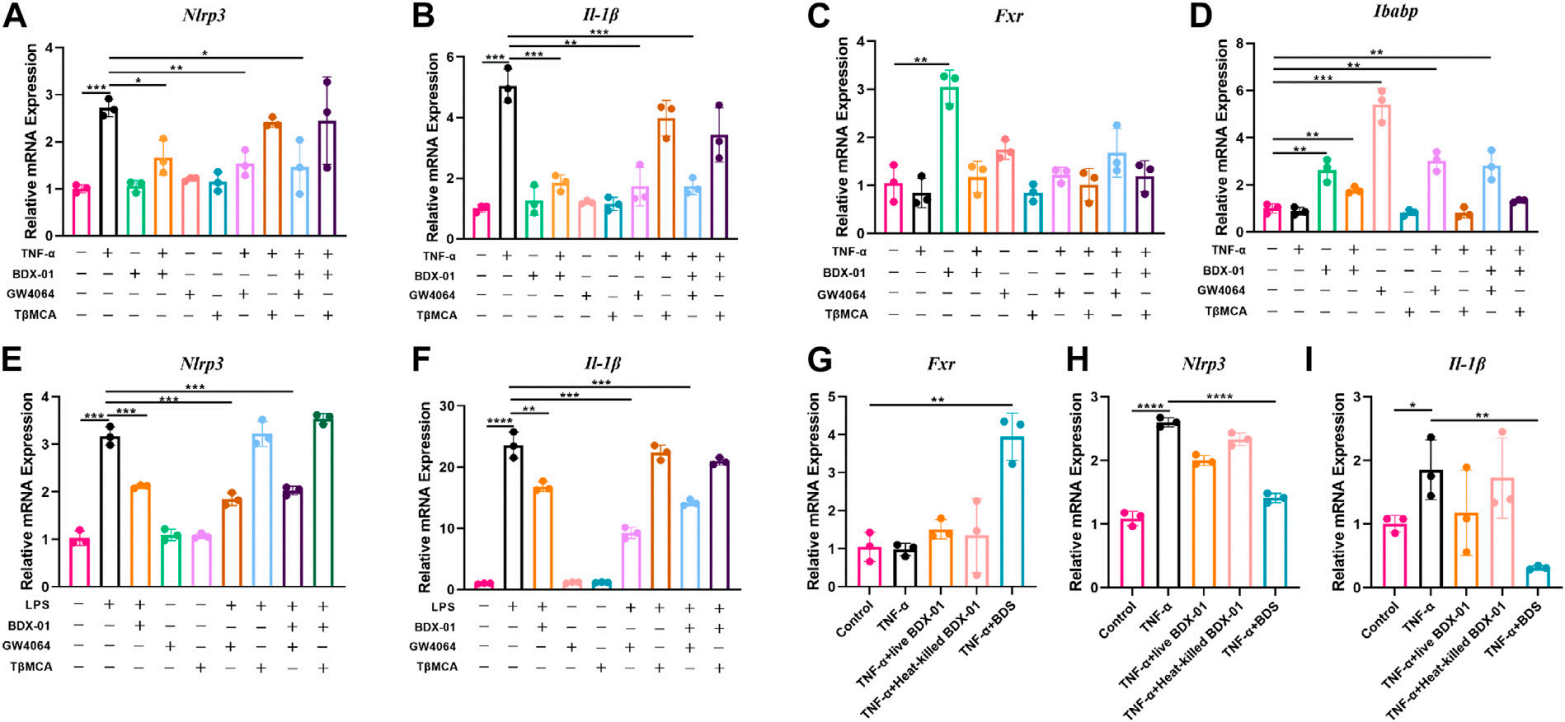
BDX-01 downregulated the NLRP3 inflammasome and proinflammatory factors in the intestinal cells and macrophages through the FXR-dependent pathway. **(A–D)** mRNA levels of *Nlrp3*, *Il-1β*, *Fxr*, and *Ibabp* in Caco2 cells. Caco2 cells were pretreated with BDX-01 (MOI = 1:100), GW4064 (10 μM), and TβMCA (100 μM) for 4 h, followed by TNF-α (100 ng/mL) for 4 h. **(E,F)** *Nlrp3*, *Il-1β*, *Fxr*, and *Ibabp* expression in macrophages pretreated with BDX-01 (MOI = 1:100), GW4064 (10 μM), and TβMCA (100 μM) for 4 h and LPS (1 μg/mL) for 4 h. **(G–I)** *Fxr*, *Nlrp3*, and *Il-1β* expression levels in Caco2 cells. The Caco2 cells were pretreated with live BDX-01 (MOI = 1:100), heat-killed BDX-01 (MOI = 1:100), and BDS (50%) for 4 h, followed by TNF-α (100 ng/mL) for 4 h. *p < 0.05; **p < 0.01; ***p < 0.001; ****p < 0.0001. *n* = 3 per group.

Enterocytes secrete cytokines and chemokines that promote macrophage and immunocyte infiltration into intestinal inflammatory sites (Kagnoff and Eckmann, 1997). In active UC, NLRP3, and IL-1β are expressed mainly in lamina propria immune cells (Ranson et al., 2018). We determined the anti-inflammatory effects of BDX-01 on intestinal cells and macrophages and function of FXR. We subjected human intestinal epithelial Caco-2 cells and mouse J774A.1 macrophages to TNF-α and LPS, respectively, with or without BDX-01, the FXR agonist GW4064, and the FXR antagonist T-βMCA. In response to the TNF-α/LPS treatment, *Nlrp3* and *Il-1β* mRNAs were dramatically upregulated compared with the control. Neither BDX-01, GW4064, nor T-βMCA alone affected *Il-1β* expression in intestinal cells (Figures 8A, B) or macrophages (Figures 8E, F). We confirmed the transcription of *Fxr* in the intestinal cells in response to BDX-01 treatment compared to GW4064 and TβMCA (Figures 8C, D). BDX-01 significantly downregulated *Nlrp3* and *Il-1β* mRNA induced by TNF-α and LPS and blocked by TβMCA (Figures 8A–F). Therefore, FXR activation counteracts the expression of the NLRP3 inflammasome and proinflammatory factor IL-1β expression in intestinal cells and macrophages.

We co-treated Caco-2 cells with TNF-α, BDX-01 culture supernatant (BDS), and live and heat-killed bacterial cells to determine the anti-inflammatory effects of the BDX-01 components. Whereas BDS treatment upregulated *Fxr* and downregulated *Nlrp3* and *Il-1β*, the bacterial cells had no such effects (Figures 8G–I).

## 4 Discussion

The role of the gut microenvironment in UC progression is attracting increasing research attention. *Bacteroides* is a dominant and biologically important commensal bacteria in the human colon, and its relative abundance increases markedly in the mucosal tissue of patients with UC (Lucke et al., 2006). However, several strains of *Bacteroides*, including *B. fragilis* NCTC 9343, *B. thetaiotaomicron* DSM 2079, and *B. cellulosilyticus* DSM 14838, can ameliorate intestinal inflammation and tissue damage associated with IBD (Mazmanian et al., 2008; Neff et al., 2016; Delday et al., 2019). Here, we showed that a novel strain of *B. dorei* (BDX-01) isolated from healthy human stool, designated as BDX-01, could alleviate DSS-induced colitis by regulating gut BA metabolism and the FXR-NLRP3 signaling pathway.

*B. dorei* was recently isolated and distinguished from *B. vulgatus* (Pedersen et al., 2013), a known IBD pathobiont. Although these species are closely related, they are negatively correlated and generically different, in terms of the epigenomic alterations they cause in hosts presenting with IBD (Ryan et al., 2020). The ameliorative or worsening effect of *B. dorei* in different disease models is also controversial (Sánchez et al., 2010; Davis-Richardson et al., 2014; Davis-Richardson and Triplett, 2015; Zhang et al., 2015; Yao et al., 2018; Yoshida et al., 2018; Song et al., 2021). Therefore, its probiotic properties may be disease specific, and its safety must be carefully evaluated.

Colonic mucosal injury in DSS-induced colitis mouse model is characterized by inflammatory cell infiltration, crypt injury, goblet cell depletion, and mucosal erosion. In addition, histological damage was closely associated with loss of body weight, rectal bleeding, and colon shortening. Treatment with BDX-01 significantly alleviated DSS-induced increases in DAI and histopathology scores. Inflammatory reactions promote the production of proinflammatory cytokines that contribute to the progression of colitis. Here, TNF-α, IL-1β, and IL-6 levels were dramatically elevated in the DSS group compared with the control. However, these changes improved in both the Mesalazine and BDX-01 groups. Moreover, the putative virulence factors in the BDX-01 genome do not contain those known to be involved in the pathogenesis of *B. dorei*. Notably, no plasmids were found in the genome, suggesting that the chances of transferring the characteristics of antibiotic resistance to other intestinal symbiotic bacteria are low. Antibiotic resistance tests revealed that BDX-01 is sensitive to cefoperazone, meropenem, clindamycin, clarithromycin, tetracycline, and ciprofloxacin. Hence, it can be easily, rapidly, and effectively eliminated when necessary. Further safety experiments confirmed that it was nontoxic and non-pathogenic both *in vitro* and *in vivo*.

Dysregulation of BA metabolism disrupts intestinal homeostasis by causing immune imbalance and dysbacteriosis, which lead to gut damage (Tiratterra et al., 2018). BA homeostasis might have been disrupted in mouse DSS-induced colitis models. Here, we found that the unconjugated/conjugated primary BA ratios and secondary BA levels were markedly decreased in mice with DSS-induced colitis. Thus, BA deconjugation and dehydroxylation were impaired. BDX-01 had unique effects on the BA profile and regulation of BA homeostasis. We observed that BDX-01 increased fecal total BA level, CA/TCA and βMCA/TβMCA ratios, and DCA level in the mouse colitis model. Therefore, it might have stimulated BA excretion, thus improving the gut deconjugation function. The gut microbiota plays an important role in regulating intestinal BA metabolism. Here, BDX-01 treatment enriched the gut microbiome with BSH-containing phyla and increased BSH activity in DSS-induced colitis mouse model. These results suggest that BDX-01 alleviated colitis by promoting the recovery of the BSH-producing microbiota and primary BA deconjugation in the colon.

Bile acids bind to different bile acid receptors to exert signaling molecular functions. Although many intestinal bile acid receptors have anti-inflammatory effects upon bile acid ligand activation (Fuchs and Trauner, 2022), their regulation is not always consistent in response to a complex bile acid spectrum. We found that BDX-01 treatment significantly upregulated intestinal FXR expression and downregulated TGR5 and VDR expression, but the combined effect was still alleviation of intestinal inflammation. Although there is some controversy regarding the rank order of the FXR-activating potentials of bile acids, there is a consensus that unconjugated bile acids have greater potential to activate FXR than conjugated bile acids, possibly due to the greater lipophilicity of unconjugated bile acids (Jia et al., 2018). TβMCA inhibited the FXR signaling pathway in the presence of FXR agonists TCA and TCDCA (Li et al., 2013). Thus, BA deconjugation may be important for upregulating FXR signaling. Our findings suggest that BDX-01 increased the CA/TCA and βMCA/TβMCA ratios and DCA level, decreasing intestinal FXR antagonism and upregulating colonic FXR. Thus we propose that BDX-01 could accumulate BA-based activators, upregulate colonic FXR, and inhibit the intestinal inflammation.

FXR activation has been reported to reduce proinflammatory cytokine expression through inhibition of NF-κB activity and inflammasome assembly of NLRP3 (Chen et al., 2019). The NLRP3 inflammasome is ubiquitous among epithelial cells and immunocytes (Fusco et al., 2020). It is an innate immune receptor that binds to microbial ligands to mediate the assembly of complexes of inflammasomes, including NLRP3, ASC, and caspase-1. It triggers caspase-1 activation and IL-1β and IL-18 secretion and participates in the development of UC (Zhen and Zhang, 2019). Animal and human studies have revealed that the role of the NLRP3 inflammasome in the pathogenesis of IBD remains unclear (Thomas et al., 2022); in this study, we found that hyperactivation of intestinal NF-κB and NLRP3 aggravated experimental colitis in mice. BDX-01 downregulated NLRP3, ASC, and caspase-1 and reduced IL-1β secretion in colon tissue, but did not inhibit the activation of NF-κB. Therefore, BDX-01 inhibits the excessive activation of NLRP3 inflammasome through the FXR-NLRP3 pathway, and play an anti-inflammatory effect.

**FIGURE 9:**
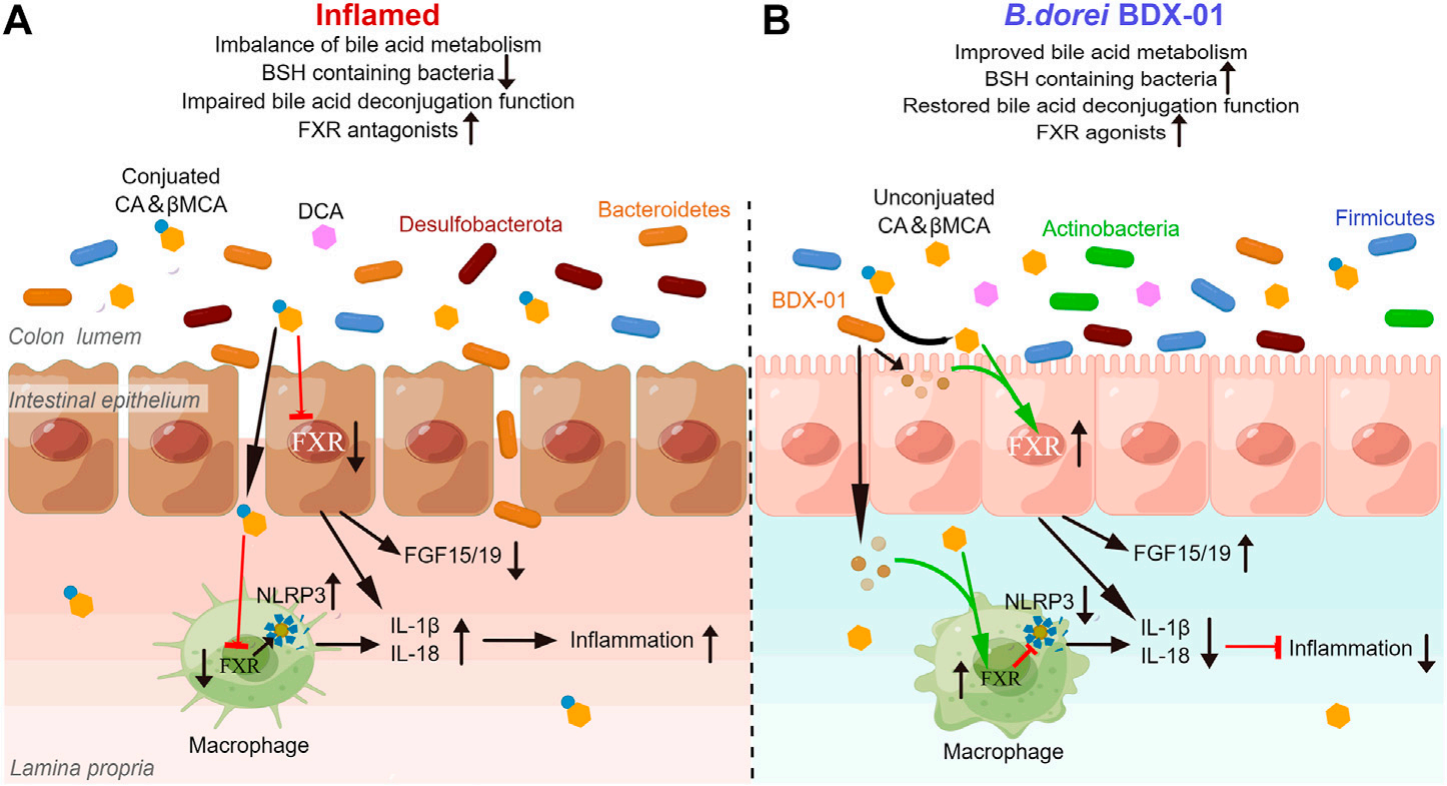
BDX-01 alleviates colitis by regulating the FXR/NLRP3 inflammasome signaling pathways in intestinal mucosal barriers by modulating the gut microbiota and bile acids. **(A)** During colitis progression, an imbalance in the gut microbiota leads to the invasion of pathogenic bacteria, such as Proteobacteria, intestinal barrier damage, macrophage infiltration, activation of intestinal epithelial cells and the macrophage inflammasome signaling pathway, and proinflammatory cytokine production. Reduction in intestinal BSH-producing bacteria alters BA unconjugation and lowers the unconjugated/conjugated primary BA ratios. Increases in conjugated BA, such as TβMCA, inhibit the farsenoid X receptor (FXR). FXR in intestinal epithelial cells and macrophages is inhibited in a BA-dependent manner, texacerbating NLRP3 inflammasome activation. **(B)** In the treatment of colitis, BDX-01 improves gut dysbiosis, promotes the recovery of BSH-producing bacteria, such as Firmicutes and Actinobacteria, restores gut deconjugation, increases unconjugated/conjugated primary BA ratios, and activates FXR. BDX-01 and its metabolites directly activate FXR. FXR activation in intestinal epithelial cells and macrophages suppresses the NLRP3 inflammasome activation, downregulate the proinflammatory factors, and ameliorate colitis.

Gut bacteria contribute to the balance between BA-based FXR agonists and antagonists by metabolizing BA. Here, we noticed the effect of BDX-01 on BA levels that promote or inhibit FXR in mice. The enhancing effect of BDX-01 on fecal BSH activity was abolished when gut microbiota was depleted by Abx, but BDX-01 still significantly alleviated colitis and upregulated colonic FXR activity. This suggests that in addition to altering BSH activity, more importantly, BDX-01 itself activates FXR in a BA-independent manner. Subsequent *in vitro* cell experiments showed that the FXR agonist was derived from the culture supernatant of BDX-01. BDX-01 also inhibited the activation of the NLRP3 inflammatory signaling pathway and mitigated damage to TNF-α/LPS-treated cells. However, when cells were co-treated with TβMCA, the beneficial effects of BDX-01 were attenuated. Therefore, in addition to increasing the abundance of BSH-producing bacteria, BDX-01 might directly activate FXR through unknown metabolites and mechanisms (Figure 9).

The effects of BDX-01 treatment on intestinal injury and inflammation resembled those of mesalazine treatment. Neither α-diversity nor β-diversity significantly differed between the Mesalazine and DSS groups. However, the mesalazine group could significantly increase the abundance of *Akkermansia* and *Lachnoclostridium*, two beneficial bacteria negatively correlated with UC activity (Wang et al., 2018; Bian et al., 2019). In addition, mesalazine significantly reduced the levels of conjugated primary BAs in the gut and partially reversed the inhibition of colonic FXR activity. These findings were partially consistent with those previously reported (Huang et al., 2022). Compared with mesalazine, BDX-01 more strongly regulates the structure of the intestinal microbiota containing BSH and improves BA metabolism. Furthermore, these effects are not constrained by the gut microbiota. Therefore, BDX-01 might be more effective than mesalazine at regulating intestinal BA metabolism and, therefore, improving colitis.

This study had some limitations. First, the acute DSS colitis model is useful for analyzing innate immune mechanisms in the context of colitis (Wirtz et al., 2017), we will further explore the effect of BDX-01 on adaptive immunity with the help of other IBD models. Second, in the future study, we need to identify the active components in BDX-01 that confer the capacity to treat colitis. In this manner, we can clarify the molecular mechanisms of BDX-01 and establish its clinical applications.

Overall, this study empirically demonstrated that BDX-01 alleviates DSS-induced colitis by activating colonic FXR and inhibiting the NLRP3 inflammasome signaling pathway. More specifically, BDX-01 activates colonic FXR by direct activation, regulating intestinal BA metabolism, and increasing the abundances of BSH-containing gut bacteria. These results suggest that BDX-01 is a promising probiotic in the treatment of colitis.

## Data Availability

The datasets presented in this study can be found in online repositories. The names of the repository/repositories and accession number(s) can be found below: https://www.ncbi.nlm.nih.gov/bioproject/PRJNA900409/.
