# Supplementary material for: Bacteroides dorei BDX-01 alleviates DSS-induced experimental colitis in mice by regulating intestinal bile salt hydrolase activity and the FXR-NLRP3 signaling pathway
Source: Front Pharmacol. 2023 May 24;14:1205323. doi: 10.3389/fphar.2023.1205323 (PMC10244678; doi:10.3389/fphar.2023.1205323)
Supplement: Supplementary file 1 [file DataSheet1.docx]

**Supplementary materials**

**Supplementary Figure 1** The microbiota was depleted effectively after the antibiotic cocktail treatment.


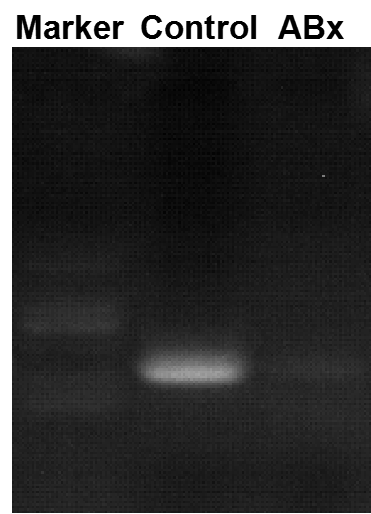


**Supplementary Figure 2** Differential abundance of bacterial OTUs in BDX-01 and DSS group at phylum levels.


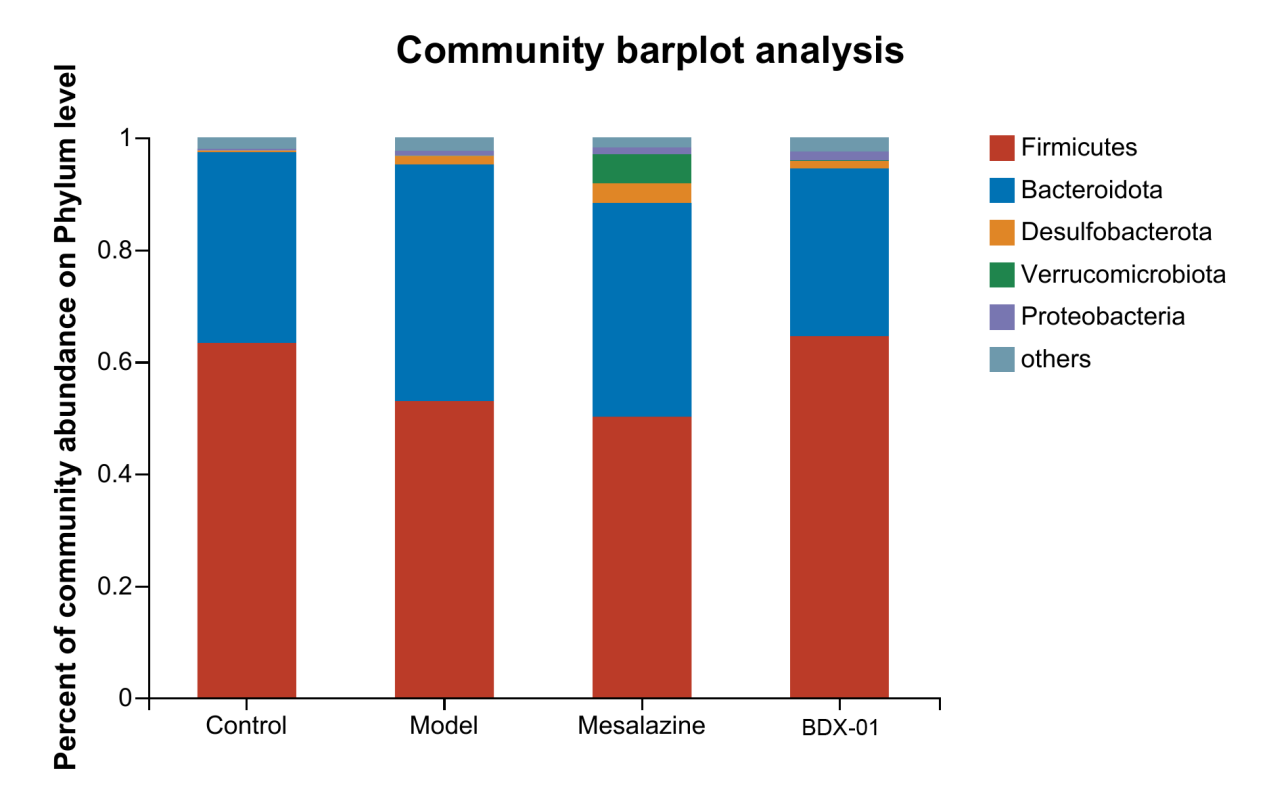


**Supplementary Figure 3** Differential abundance of bacterial OTUs in BDX-01 and DSS group at family levels.


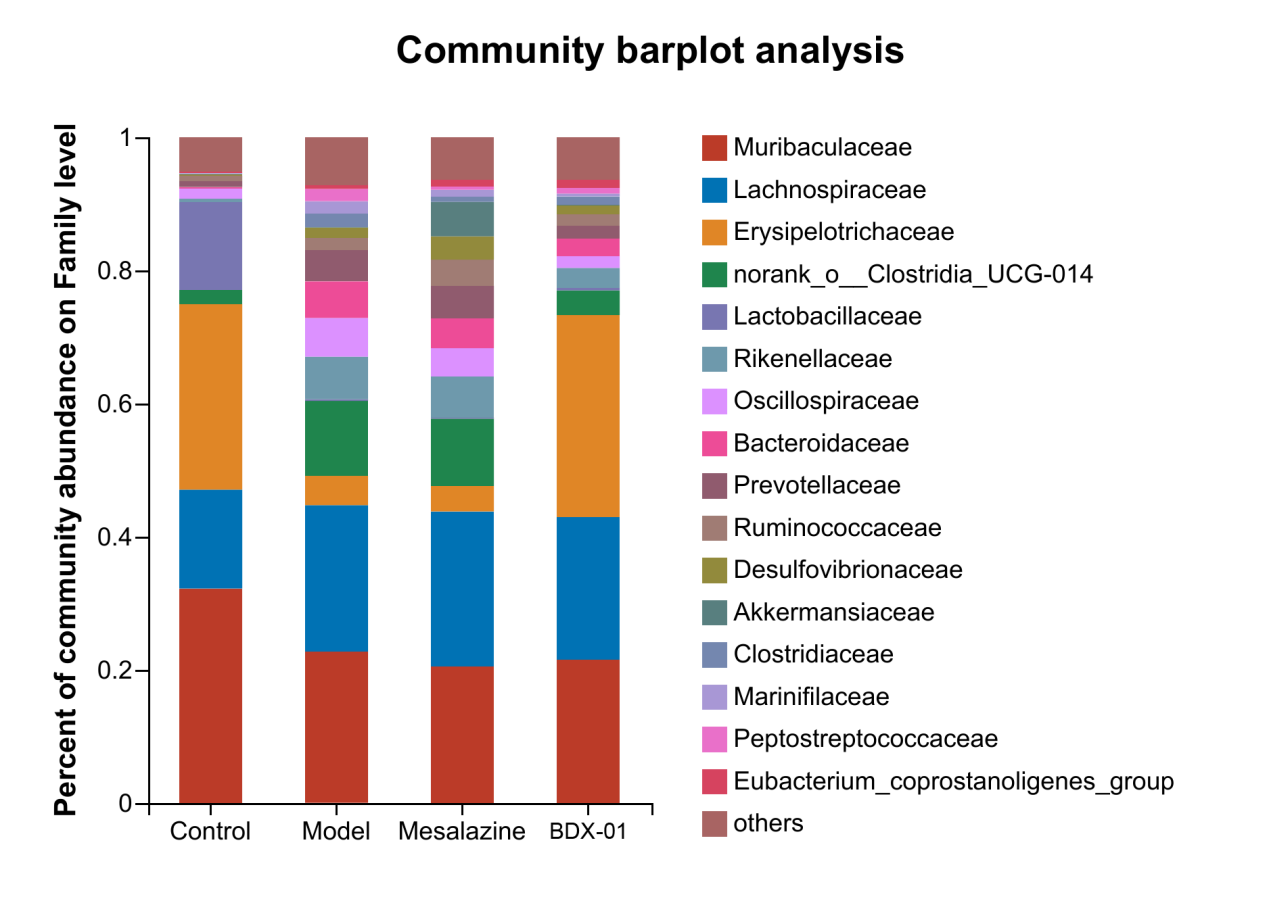


**Supplementary Figure 4** Differential abundance of bacterial OTUs in BDX-01 and DSS group at genus levels.


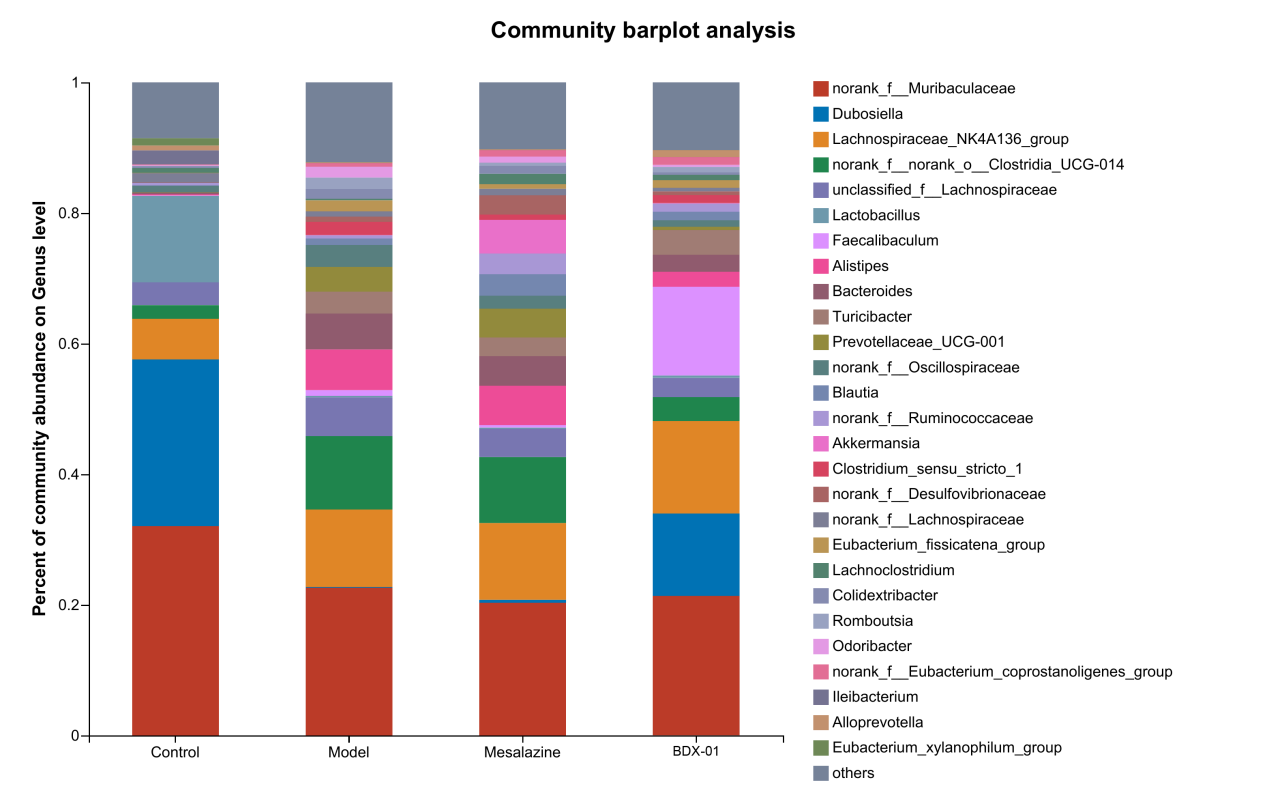


**Supplementary Figure 5** BSH activity assay of BDX-01 *in vitro.*


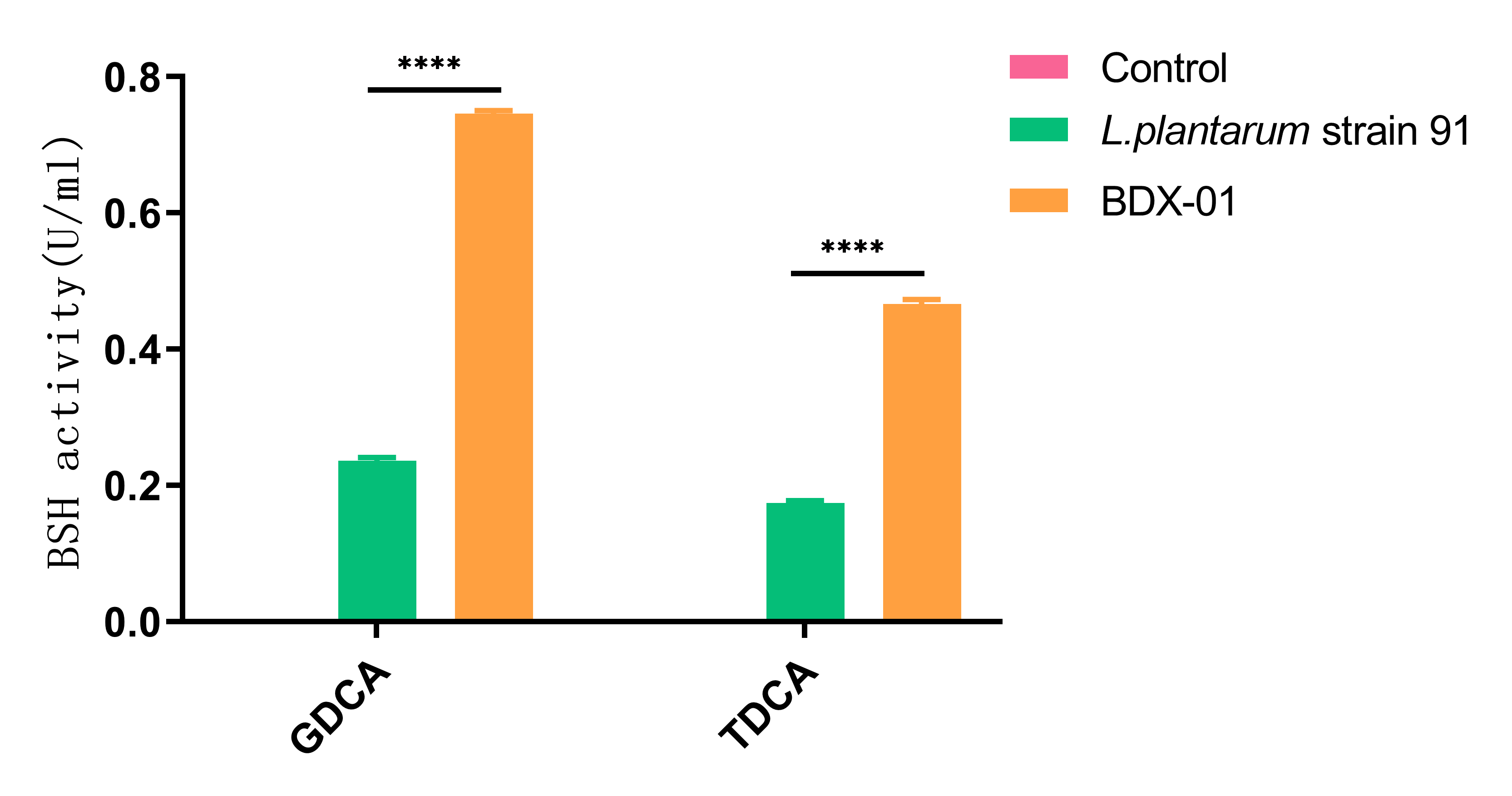


**Supplementary Figure 6** The expression of different colonic bile acid receptors were determined using western blotting analysis.


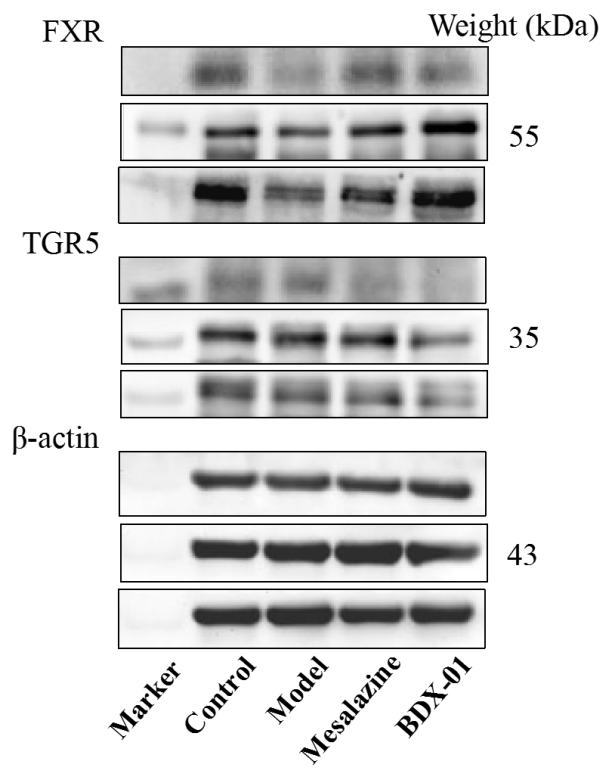

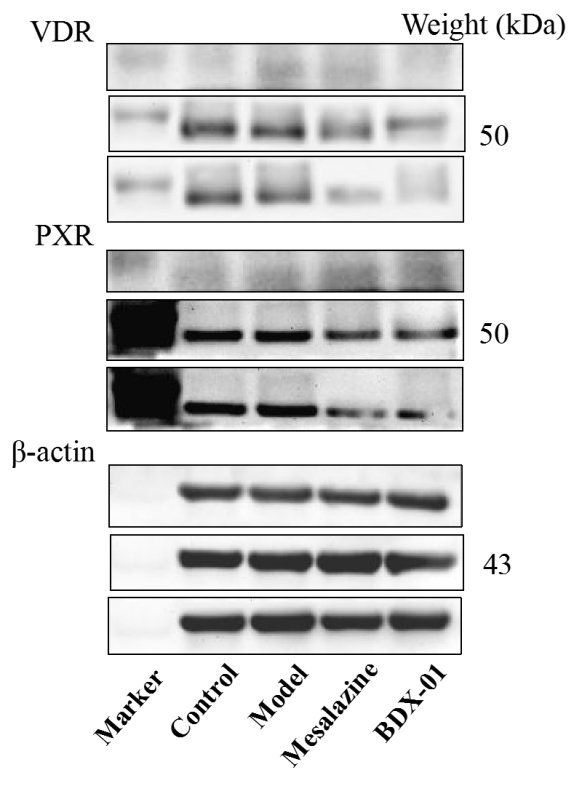


**Supplementary Figure 7** The expression of NF-κB, NLRP3, ASC, caspase-1, gsdmd, and IL-1β expression levels in different DSS groups were determined using western blotting analysis.


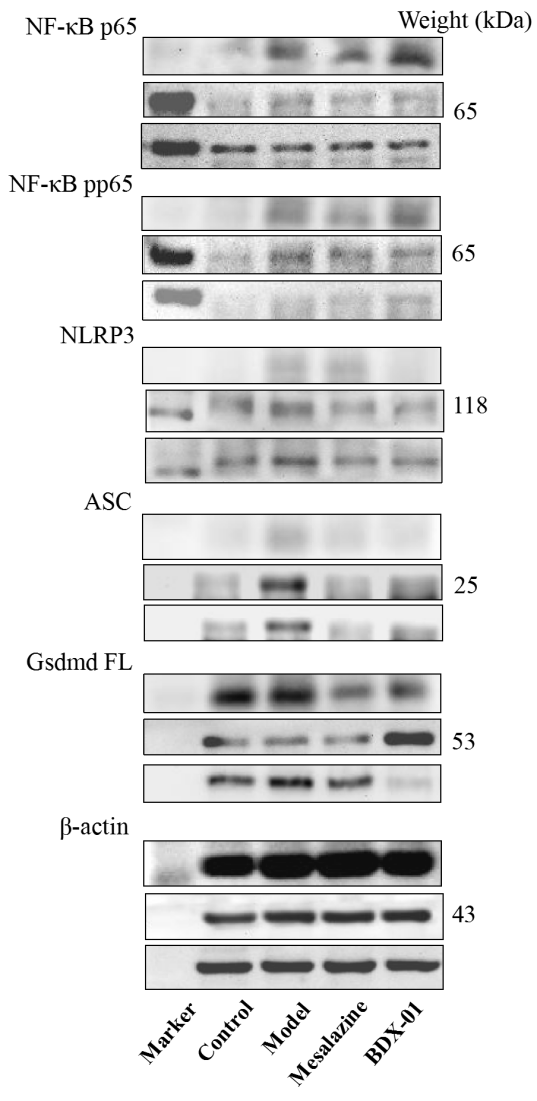

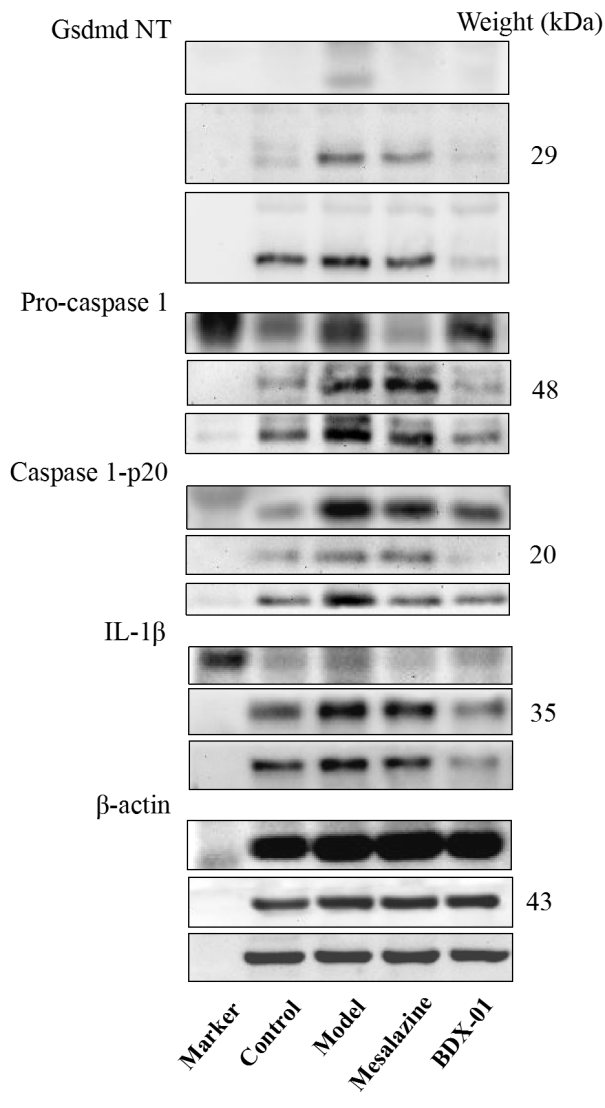


**Supplementary Figure 8** The expression of FXR, FGF15, NLRP3, and IL-1β expression levels in different DSS groups following antibiotic treatment were determined using western blotting analysis.


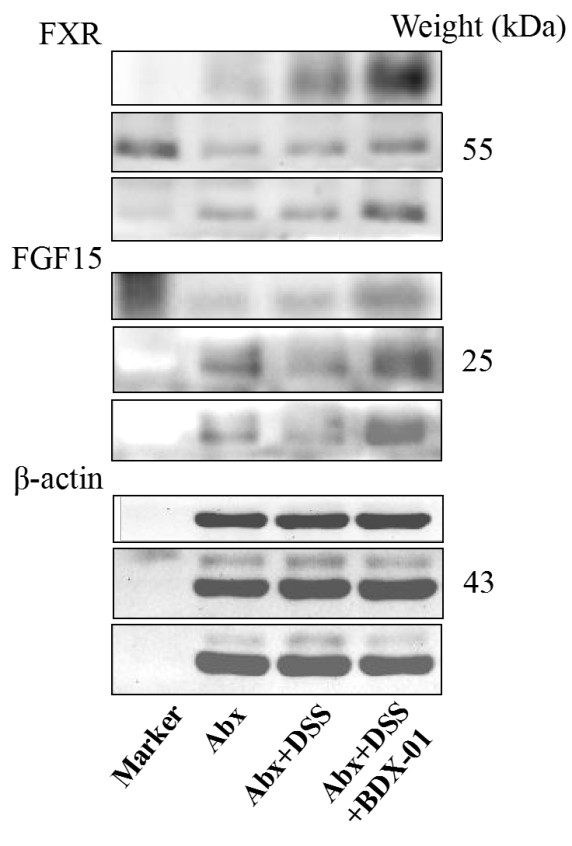

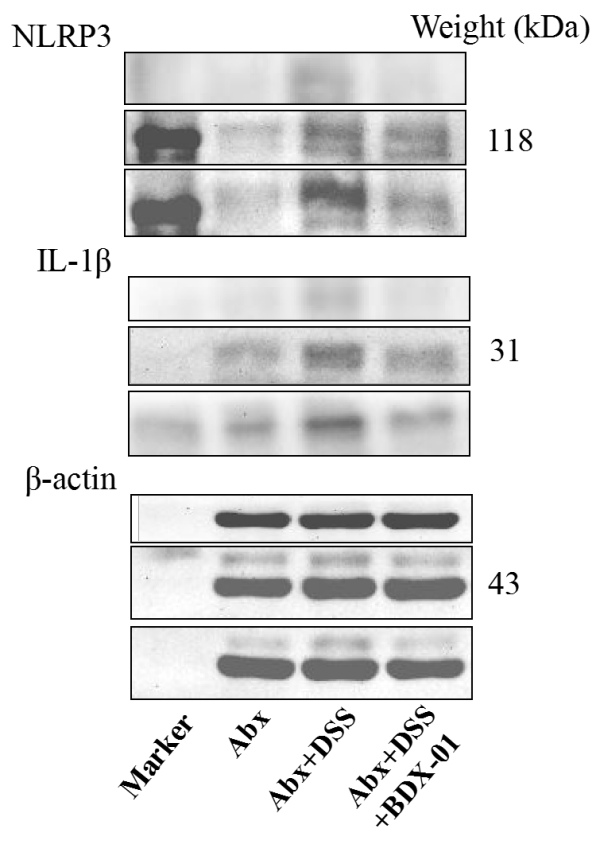


**Supplementary Table 1** Calculated disease activity index (DAI) score.

| **Score** | **Weight loss (%)** | **Stool consistency** | **Blood in feces** |
| --- | --- | --- | --- |
| 0 | None | Normal | Negative (no bleeding) |
| 1 | 1.0-5.0 | ­ | ­ |
| 2 | 5.0-10.0 | Loose stools | Positive (slight bleeding) |
| 3 | 10.0-15.0 | ­ | ­ |
| 4 | Over 15.0 | Watery diarrhea | Gross bleeding |

**Supplementary Table 2** Histological scores of dextran sulfate sodium (DSS)-induced colitis.

| **Histological features** | **Score** | **Description** |
| --- | --- | --- |
| **Epithelium loss** | 0 | None |
|  | 1 | 0.0-5.0% |
|  | 2 | 5.0-10.0% |
|  | 3 | Over 10.0% |
| **Crypt damage** | 0 | None |
|  | 1 | 0.0-10.0% |
|  | 2 | 10.0-20.0% |
|  | 3 | Over 20.0% |
| **Depletion of goblet cells** | 0 | None |
|  | 1 | Mild |
|  | 2 | Moderate |
|  | 3 | Severe |
| **Infiltration of inflammatory cells** | 0 | None |
|  | 1 | Mild |
|  | 2 | Moderate |
|  | 3 | Severe |

**Supplementary Table 3 Primer sequences for qRT-PCR analysis.**

| Gene | Forward Primer Sequence | Reverse Primer Sequence |
| --- | --- | --- |
| FXR | TGACCTGTGAGGGGTGTAAA | GTCGACACTCTTGACACTTT |
| IBABP | TCACTTGGTCCCAGCACTA | CTTGTCACCCACGATCTCT |
| IL-1β | ATGATGGCTTATTACAGTGGCAA | GTCGGAGATTCGTAGCTGGA |
| NLRP3 | CTTCCTTTCCAGTTTGCTGC | TCTCGCAGTCCACTTCCTTT |
| GAPDH | GAAGGTGAAGGTCGGAGT | CATGGGTGGAATCATATTGGAA |
| Tnf-α | CGTGCTCCTCACCCACAC | GGG TTCATACCAGGGTTTGA |
| Il-1β | TCAGGCAGGCAGTATCACTCATT | GGAAG GTCCACGGGAAAGA |
| Il-6 | TAGTCCTTCCTACCCCAATTTCC | TTGGTCCTTAGCCACTCCTTC |
| Il-10 | GCTCTTACTGACTGGCATGAG | CGCAGCTCTAGGAGCATGTG |
| Nlrp3 | AGAGCCTACAGTTGGGTGAAATG | CCACGCCTACCAGGAAATCTC |
| Caspase-1 | ATCCGTTCCATGGGTGAAGGTACA | CAAATGCCTCCAGCTCTGTAATCA |
| Gapdh | GGTTGTCTCCTGCGACTTCA | TGGTCCAGGGTTTCTTACTCC |

Genes in capitals indicate human genes, and those in lower case are mouse genes. All genes were analyzed using the following conditions: 95 °C 30s, 40 cycles of 95 °C for 5 s, and 60 °C for 30 s.

**Supplementary Table 4 Hematologic and serum biochemical analysis of mice in the acute animal toxicity experiment with BDX-01.**

Hematologic analysis

| Blood index | Control | BDX-01 | P value |
| --- | --- | --- | --- |
| [White blood cell count](javascript:;) (10^9/L) | 2.008 ± 0.722 | 2.902 ± 0.6965 | 0.4065 |
| [Neutrophil count](javascript:;) (10^9/L) | 0.065 ± 0.005 | 0.148 ± 0.06778 | 0.3167 |
| Red blood cell count (10^12/L) | 13.75 ± 1.324 | 13.62 ± 0.8755 | 0.9366 |
| Hemoglobin (g/L) | 224.3 ± 21.63 | 225.4 ± 15.26 | 0.9656 |
| Platelets (10^9/L) | 860.5 ± 132.1 | 1288 ± 404.7 | 0.3960 |

Serum biochemical analysis

| Serum biochemical index | Control | BDX-01 | P value |
| --- | --- | --- | --- |
| ALT(U/L) | 35.7 ± 4.4 | 34.02 ± 3.182 | 0.7599 |
| AST(U/L) | 160 ± 25.62 | 122.5 ± 25.33 | 0.3384 |
| CR(μmol/L) | 14.13 ± 1.314 | 14.62 ± 0.7552 | 0.7407 |
| BUN(mmol/L) | 7.445 ± 0.2886 | 7.288 ± 0.2942 | 0.7190 |
| TBA(μmol/L) | 7.445 ± 0.2886 | 4.62 ± 1.642 | 0.2359 |

**Supplementary Table 5 Detailed relative BA alterations in feces in acute colitis experiments**

| **Bile acid name** | **M1** | **M2** | **M3** | **M8** | **B2** | **B6** | **B1** | **B5** | **C1** | **C2** | **C3** | **C5** | **P1** | **P2** | **P3** | **P5** |
| --- | --- | --- | --- | --- | --- | --- | --- | --- | --- | --- | --- | --- | --- | --- | --- | --- |
| Dehydrolithocholic acid | 2038.84 | 876.54 | 1489.45 | 1748.55 | 501.20 | 1223.55 | 1117.41 | 499.60 | 3136.36 | 6740.15 | 2910.70 | 7066.67 | 3983.92 | 783.79 | 1128.10 | 904.51 |
| Isoallolithocholic acid/Isolithocholic acid | 1934.30 | 2189.71 | 2914.84 | 4643.57 | 2672.00 | 1170.25 | 5441.30 | 1578.71 | 5176.03 | 9282.24 | 6137.86 | 12823.58 | 6849.02 | 2549.01 | 2236.78 | 2986.89 |
| Lithocholic acid | 10316.53 | 10376.54 | 12398.83 | 16836.93 | 11256.80 | 13851.24 | 28313.36 | 24813.65 | 21095.45 | 35301.54 | 24164.61 | 51113.41 | 30529.80 | 10551.38 | 6791.74 | 15870.90 |
| 23-Nordeoxycholic acid | 188.43 | 860.91 | 1108.20 | 1084.65 | 99.60 | 370.66 | 572.87 | 80.72 | 102.07 | 132.43 | 318.11 | 301.22 | 149.02 | 549.80 | 1283.88 | 715.16 |
| 6-Ketolithocholic acid | 2553.31 | 1427.98 | 2805.08 | 3416.60 | 1149.20 | 2714.46 | 1984.21 | 1046.99 | 3519.42 | 12036.29 | 2108.64 | 9741.06 | 4070.98 | 1126.88 | 2457.02 | 1781.97 |
| 7-Ketolithocholic acid | 500.00 | 189.30 | 289.84 | 187.97 | 204.80 | 337.19 | 175.71 | 228.51 | 807.02 | 5637.07 | 754.32 | 1350.00 | 642.75 | 163.64 | 211.16 | 171.72 |
| 12-Ketolithocholic acid | 48866.12 | 33719.75 | 32511.33 | 36779.67 | 3529.60 | 37902.89 | 33042.91 | 11922.49 | 55269.42 | 192542.47 | 73185.19 | 128863.01 | 93657.65 | 17711.07 | 34111.57 | 19890.57 |
| Apocholic acid | 252.07 | 188.89 | 376.17 | 397.93 | 189.20 | 819.01 | 227.53 | 1478.31 | 788.43 | 1163.71 | 906.17 | 1945.12 | 680.39 | 213.83 | 347.11 | 240.57 |
| Isoursodeoxycholic acid | 328.10 | 298.35 | 113.67 | 76.35 | 302.40 | 523.97 | 361.94 | 784.34 | 1080.17 | 5497.30 | 1190.95 | 3086.59 | 1344.71 | 229.64 | 130.17 | 355.33 |
| Murideoxycholic acid | 3127.27 | 1855.56 | 2628.13 | 3256.43 | 1750.00 | 3709.09 | 4674.09 | 5443.37 | 4078.93 | 12233.59 | 2707.41 | 8202.85 | 6023.92 | 1467.19 | 1276.03 | 1295.08 |
| Isohyodeoxycholic acid | 4666.12 | 4081.89 | 5900.00 | 10904.98 | 5125.20 | 5212.40 | 7405.26 | 6965.46 | 6591.74 | 14885.33 | 4097.94 | 15015.45 | 8110.98 | 2946.25 | 2322.73 | 2345.49 |
| Ursodeoxycholic acid | 323.97 | 374.90 | 369.14 | 235.27 | 623.60 | 1094.21 | 398.79 | 1874.70 | 2823.97 | 10979.92 | 3542.80 | 5188.62 | 1272.94 | 404.74 | 195.45 | 967.62 |
| Hyodeoxycholic acid | 27597.93 | 21441.15 | 29548.83 | 49943.15 | 27930.00 | 42211.16 | 46921.05 | 63392.77 | 34746.28 | 75993.82 | 18293.42 | 74364.63 | 41998.82 | 15318.58 | 9808.26 | 14287.30 |
| 3-Epideoxycholic acid | 9885.95 | 12085.19 | 11606.64 | 13965.98 | 2615.60 | 12344.63 | 16185.83 | 23517.27 | 11463.22 | 32889.96 | 15536.63 | 28652.85 | 16626.67 | 8042.29 | 8011.16 | 11169.67 |
| Chenodeoxycholic acid | 3938.43 | 5847.33 | 8994.14 | 11870.12 | 4377.60 | 16603.31 | 8619.84 | 18814.46 | 7447.93 | 30182.63 | 15639.92 | 29030.89 | 8794.12 | 7342.69 | 6571.49 | 8558.61 |
| Deoxycholic acid | 175669.42 | 216925.10 | 189842.97 | 230575.10 | 49645.60 | 293764.05 | 398242.11 | 518690.76 | 193372.73 | 613949.42 | 332916.87 | 665192.68 | 354784.71 | 138360.87 | 170297.11 | 236032.79 |
| Isodeoxycholic acid | ND | ND | ND | ND | ND | ND | ND | ND | ND | ND | ND | ND | ND | ND | ND | ND |
| Nor Cholic Acid | 548.35 | 287.65 | 541.41 | 280.50 | 985.60 | 1948.76 | 1033.60 | 1397.19 | 1045.87 | 925.10 | 724.28 | 1031.30 | 1166.27 | 352.17 | 260.74 | 780.74 |
| Dehydrocholic acid | 36.36 | 12.76 | 20.31 | 20.33 | 7.20 | 19.01 | 8.10 | 21.69 | 20.25 | 255.60 | 33.74 | 47.15 | 35.29 | 12.25 | 21.90 | 34.02 |
| 7,12-Diketolithocholic acid | 178.10 | ND | 72.27 | ND | 73.60 | 138.02 | ND | 130.12 | 308.68 | 7241.31 | 227.57 | 675.20 | 225.49 | 30.43 | 82.64 | 51.23 |
| 6,7-Diketolithocholic acid | 183.47 | 351.03 | 412.50 | 468.46 | 218.80 | 385.54 | 404.86 | 553.82 | 570.25 | 1489.58 | 418.11 | 776.42 | 487.84 | 373.52 | 373.14 | 261.48 |
| 7-Ketodeoxycholic acid | 1716.53 | 839.92 | 1014.45 | 885.48 | 3797.20 | 5796.69 | 951.42 | 4128.11 | 3852.07 | 126792.66 | 3535.80 | 8015.04 | 3697.25 | 1598.42 | 2561.16 | 3238.93 |
| 12-Dehydrocholic acid | 496.69 | 160.91 | 109.38 | 43.15 | 249.20 | 668.18 | 107.69 | 644.58 | 514.46 | 29596.14 | 527.16 | 814.63 | 454.12 | 194.47 | 238.43 | 384.84 |
| 3-Dehydrocholic acid | 919.42 | 388.89 | 479.30 | 451.04 | 883.60 | 2269.42 | 321.46 | 1669.08 | 1162.81 | 42754.83 | 1203.70 | 1886.59 | 1254.90 | 643.48 | 586.78 | 926.23 |
| Ursocholic acid | 809.92 | 1304.94 | 1426.95 | 1458.51 | 898.40 | 661.57 | 2400.40 | 536.55 | 560.74 | 988.80 | 314.40 | 996.75 | 878.04 | 1366.80 | 1338.84 | 891.39 |
| ω-Muricholic Acid | 107596.28 | 127459.26 | 202263.67 | 250269.71 | 72728.40 | 197047.93 | 280714.57 | 228422.89 | 341112.40 | 1026693.82 | 406458.02 | 867149.59 | 334545.10 | 78334.39 | 68130.17 | 116973.36 |
| 3β-Cholic Acid | ND | ND | 282.81 | 108.30 | ND | 1714.88 | ND | 2834.54 | ND | 12198.84 | ND | ND | ND | 117.00 | 90.91 | ND |
| α-Muricholic acid | 7861.57 | 5887.24 | 12805.47 | 12345.64 | 9514.40 | 41269.01 | 22460.73 | 87663.05 | 51761.16 | 283058.69 | 60422.22 | 155959.35 | 72622.75 | 5812.65 | 4092.98 | 12263.52 |
| β-Muricholic acid | 44765.70 | 21108.64 | 63040.23 | 56325.31 | 36823.60 | 115585.95 | 22579.35 | 150659.44 | 93650.83 | 382640.93 | 143611.93 | 183364.23 | 129932.16 | 26685.77 | 20978.51 | 49086.48 |
| Hyocholic acid | 207.85 | 547.74 | 1712.50 | 999.59 | 295.60 | 3339.67 | 2376.52 | 2346.59 | 2972.73 | 18957.92 | 4393.00 | 8785.77 | 2504.71 | 470.36 | 907.44 | 1663.52 |
| Allocholic acid | 1480.17 | 1275.72 | 1135.16 | 1111.62 | 2691.60 | 16683.88 | 1362.75 | 37378.71 | 2240.91 | 73730.50 | 1347.74 | 9217.07 | 2745.49 | 2363.24 | 1494.21 | 1637.30 |
| Cholic acid | 8973.14 | 7033.74 | 4360.55 | 4124.90 | 9021.20 | 23295.04 | 8731.98 | 19647.39 | 8448.76 | 237519.31 | 6934.57 | 13630.89 | 10136.08 | 12283.00 | 7076.03 | 14534.84 |
| Glycolithocholic acid | ND | ND | ND | ND | ND | ND | ND | ND | ND | ND | ND | ND | ND | ND | ND | ND |
| Glycoursodeoxycholic acid | ND | 18.93 | ND | ND | ND | ND | ND | ND | ND | 19.31 | ND | 17.89 | 21.57 | ND | ND | ND |
| Glycohyodeoxycholic acid | 14.05 | 9.05 | 13.67 | 9.13 | ND | 54.96 | 31.58 | 26.51 | 26.86 | 47.88 | ND | 50.81 | 22.75 | ND | ND | ND |
| Glycochenodeoxycholic acid | 33.88 | 39.51 | 31.25 | 42.74 | 8.80 | 26.45 | 74.90 | 38.96 | 73.55 | 103.47 | 46.09 | 86.18 | 74.12 | 37.55 | 9.92 | 20.49 |
| Glycodeoxycholic acid | 48.76 | 47.33 | 33.20 | 26.97 | 40.40 | 170.25 | 104.86 | 136.55 | 81.40 | 201.54 | 99.59 | 141.87 | 58.82 | 38.74 | 39.67 | 77.87 |
| Lithocholic Acid-3-Sulfate | 106.61 | 103.29 | 74.61 | 79.67 | 163.60 | 109.92 | 79.76 | 176.31 | 135.12 | 78.38 | 37.04 | 54.88 | 103.92 | 73.12 | 56.61 | 79.51 |
| Glycodehydrocholic acid | 8.26 | ND | ND | 39.42 | 24.00 | 23.97 | ND | 43.37 | ND | ND | ND | ND | ND | ND | ND | 20.49 |
| Glycohyocholic acid | ND | ND | ND | ND | ND | ND | ND | ND | ND | ND | ND | ND | ND | ND | ND | ND |
| Glycocholic acid | 32.64 | ND | ND | ND | ND | ND | ND | 27.31 | ND | 89.58 | 25.10 | ND | ND | ND | ND | ND |
| Ursodeoxycholic acid 3-Sulfate | 982.64 | 1662.96 | 1496.88 | 1773.03 | 1212.40 | 1024.38 | 1252.63 | 978.31 | 1949.17 | 927.03 | 1550.62 | 2246.34 | 610.59 | 1106.32 | 1593.39 | 527.46 |
| Chenodeoxycholic Acid-3-Sulfate | 147.11 | 253.50 | 227.34 | 290.46 | 135.60 | 204.55 | 278.95 | 179.52 | 202.07 | 119.31 | 83.13 | 145.12 | 210.20 | 104.35 | 182.23 | 129.51 |
| Deoxycholic Acid-3-Sulfate | 195.04 | 190.12 | ND | ND | ND | ND | ND | ND | ND | ND | ND | ND | ND | 179.84 | ND | 156.15 |
| Taurolithocholic acid | ND | ND | ND | ND | ND | ND | ND | ND | ND | ND | ND | ND | ND | ND | ND | ND |
| Cholic Acid-3-Sulfate | 6472.73 | 4082.72 | 3432.81 | 12662.24 | 719118.80 | 374871.90 | 15458.70 | 309040.56 | 370477.69 | 562510.04 | 253737.86 | 622284.15 | 278329.80 | 39971.94 | 50076.45 | 11580.33 |
| Tauroursodeoxycholic acid | 65.29 | 49.38 | 83.20 | 50.62 | 158.40 | 66.53 | 23.08 | 104.42 | 278.10 | 672.59 | 297.12 | 405.28 | 89.02 | 65.22 | 29.75 | 80.33 |
| Taurohyodeoxycholic acid | 58.26 | 41.56 | 44.92 | 56.43 | 133.60 | 105.79 | 119.84 | 128.11 | 500.41 | 825.87 | 100.41 | 423.17 | 115.29 | 33.20 | 27.27 | 25.00 |
| Taurochenodeoxycholic acid | 59.09 | 60.08 | 110.16 | 119.50 | 116.00 | 116.12 | 91.90 | 233.73 | 675.21 | 1379.92 | 424.28 | 552.44 | 212.94 | 95.65 | 82.64 | 88.11 |
| Taurodeoxycholic acid | 271.49 | 372.43 | 166.02 | 107.88 | 165.60 | 611.57 | 870.04 | 636.14 | 282.64 | 2685.33 | 336.21 | 536.59 | 574.90 | 176.28 | 106.61 | 141.39 |
| Glycolithocholic Acid-3-Sulfate | 64.05 | 91.77 | 83.98 | 70.95 | 51.60 | 45.45 | 40.49 | 39.76 | 100.41 | 74.90 | 205.35 | 83.74 | 47.45 | 173.52 | 78.51 | 43.03 |
| Tauro ω-muricholic acid | 335.95 | 392.59 | 419.53 | 305.39 | 693.60 | 509.92 | 540.89 | 522.49 | 923.14 | 5793.05 | 886.83 | 1260.16 | 474.12 | 217.39 | 171.90 | 239.75 |
| Tauro α-Muricholic acid | 118.18 | 79.01 | 168.75 | 85.89 | 280.00 | 271.49 | 124.70 | 538.15 | 921.90 | 3753.67 | 986.01 | 1131.71 | 307.84 | 50.59 | 53.31 | 161.89 |
| Tauro β-Muricholic acid | 1058.68 | 1067.08 | 1368.75 | 855.19 | 2065.20 | 920.25 | 307.29 | 1200.80 | 2792.98 | 7059.85 | 3276.13 | 2369.11 | 843.92 | 523.72 | 457.02 | 966.39 |
| Taurohyocholic acid | ND | ND | ND | ND | ND | ND | ND | ND | ND | 148.65 | ND | ND | ND | ND | ND | ND |
| Taurocholic acid | 677.69 | 909.47 | 816.80 | 498.76 | 543.60 | 949.17 | 545.75 | 1183.13 | 1700.00 | 4408.49 | 2447.33 | 2104.88 | 857.25 | 462.45 | 483.47 | 655.33 |
| Glycoursodeoxycholic Acid-3-Sulfate | ND | ND | ND | ND | 34.00 | ND | ND | ND | ND | ND | ND | ND | ND | ND | ND | ND |
| Glycochenodeoxycholic Acid 3-Sulfate | 115.29 | 130.86 | 167.58 | 171.37 | 209.60 | 278.51 | 288.66 | 236.14 | 254.55 | 551.74 | 205.35 | 233.74 | 104.31 | 109.09 | 118.60 | 156.97 |
| Glycodeoxycholic Acid-3-Sulfate | 170.66 | 235.80 | 277.73 | 390.87 | 707.20 | 395.45 | 557.09 | 267.87 | 814.46 | 957.92 | 599.59 | 923.98 | 626.67 | 483.79 | 67.77 | 329.51 |
| Glycocholic Acid-3-Sulfate | ND | ND | ND | ND | 66.80 | ND | ND | ND | 45.45 | 41.70 | ND | 43.09 | ND | 48.22 | ND | ND |
| Taurolithocholic Acid-3-Sulfate | 26.03 | 29.22 | 141.80 | 129.46 | 55.20 | 61.57 | 58.30 | 50.20 | 191.74 | 49.81 | 103.70 | 44.72 | 74.12 | 117.00 | 103.31 | 26.23 |
| Chenodeoxycholic acid-3-β-D-Glucuronide | ND | ND | 12.11 | 14.94 | 10.00 | 11.16 | ND | 10.44 | ND | 11.97 | ND | 12.60 | 10.98 | 20.55 | ND | 11.48 |
| Chenodeoxycholic acid 24-Acyl-β-D-glucuronide | 63.64 | 47.74 | 75.78 | 82.57 | 32.00 | 64.46 | 92.31 | 31.73 | 112.40 | 120.46 | 130.04 | 57.32 | 54.90 | 43.87 | ND | 34.43 |
| Tauroursodeoxycholic Acid-3-Sulfate | ND | ND | 33.20 | ND | ND | ND | ND | 36.95 | ND | 32.43 | ND | 50.81 | ND | 67.19 | ND | ND |
| Taurochenodeoxycholic Acid-3-Sulfate | ND | ND | ND | ND | ND | ND | ND | ND | ND | ND | ND | ND | ND | ND | ND | ND |
| Taurodeoxycholic Acid-3-Sulfate | ND | ND | ND | ND | 428.80 | ND | ND | ND | ND | 122.39 | ND | ND | ND | ND | ND | ND |
| Taurocholic Acid-3-Sulfate | ND | 23.05 | ND | ND | 62.40 | 29.34 | ND | ND | ND | 18.53 | 27.98 | 28.86 | 26.67 | 114.62 | ND | 25.41 |
| Glycodeoxycholic acid-3-O-β-glucuronide | ND | ND | ND | ND | 22.40 | ND | ND | ND | ND | ND | ND | ND | ND | ND | ND | ND |
| Glycochenodeoxycholic Acid-3-O-β-glucuronide | ND | ND | ND | ND | ND | ND | ND | ND | ND | ND | ND | ND | ND | 18.18 | ND | ND |

Remarks: (1) Abbreviations: M-Model; B-BDX-01; C-Control; P- Positive (mesalazine)

1. Measurement concentration unit nmol/kg
2. Cell "ND" indicates no detection
